# Total Synthesis, Structure Elucidation, and Bioactivity Evaluation of the Cyclic Lipopeptide Natural Product Paenilipoheptin A

**DOI:** 10.1021/acs.orglett.5c00232

**Published:** 2025-03-18

**Authors:** Vladyslav Lysenko, Nataliia V. Machushynets, Jisca L. van Dam, Fabienne A. C. Sterk, Alexander Speer, Arthur F. J. Ram, Cornelis J. Slingerland, Gilles P. Van Wezel, Nathaniel I. Martin

**Affiliations:** † Biological Chemistry Group, Institute of Biology, Leiden University, Sylviusweg 72, 2333 BE, Leiden, The Netherlands; ‡ Molecular Biotechnology Group, Institute of Biology, Leiden University, Sylviusweg 72, 2333 BE, Leiden, The Netherlands; § Fungal Genetics and Biotechnology Group, Institute of Biology, Leiden University, Sylviusweg 72, 2333 BE, Leiden, The Netherlands; ∥ Department of Medical Microbiology and Infection Prevention, Amsterdam University Medical Centre, 1081 HZ, Amsterdam, The Netherlands; # Department of Microbial Ecology, Netherlands Institute of Ecology, 6700 PB, Wageningen, The Netherlands

## Abstract

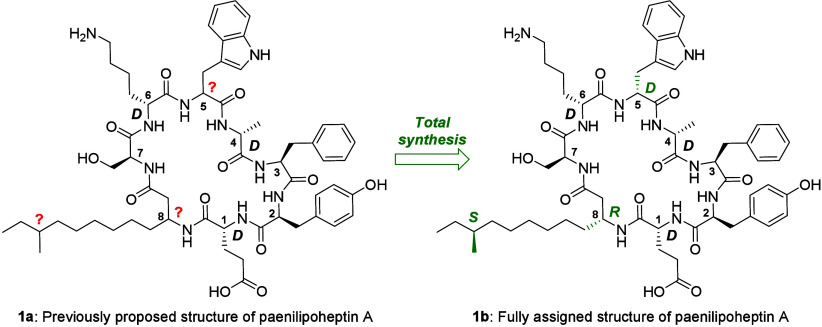

In this study, we further investigated the structure of the recently reported cyclic lipopeptide natural product paenilipoheptin A. Here, we disclose the first total synthesis of the compound, allowing for its complete structural assignment. The route developed employs automated SPPS, providing access to the compound in quantities suitable for antibacterial and antifungal testing. These studies unequivocally establish the stereochemical framework of paenilipoheptin A and further reveal that the compound possesses moderate activity against Gram-positive bacteria.

The growing threat posed by antibiotic resistance has sparked greater efforts toward the discovery and development of novel antibacterial compounds.^1,2^ Many known antibiotics are derived from natural sources,^3,4^ and there is reason to believe that more are still waiting to be discovered.^5−7^ Soil-dwelling bacteria represent an important source of antibiotics, among which members of the *Paenibacillus* genus have yielded a variety of lipopeptides with a range of activities, including polymyxins, tridecaptins, paenibacterins, and fusaricidins.^8−14^ Recently, a new class of cyclic lipopeptides termed the paenilipoheptins were detected in fermentations of *Paenibacillus*.^15,16^ However, given their low production levels, the biological activities of the paenilipoheptins could not be fully established, and only partial structural assignments were possible based on genome-based predictions, Marfey’s analysis, NMR spectroscopy, and mass spectrometry-based methods.^15,16^ Given our prior experience in the synthesis and structural characterization of various cyclic- and lipopeptide antibiotics,^17−22^ the paenilipoheptins presented an interesting new family to study, with our attention particularly drawn to paenilipoheptin A (Figure 1). While a structure of paenilipoheptin A in which the connectivity of the constituent amino acids was recently proposed,^16^ a number of stereochemical ambiguities remained. This prompted us to pursue the total synthesis of paenilipoheptin A to both fully establish its structure and provide quantifies of material suitable for evaluating the compound’s biological activity.

**Figure 1:**
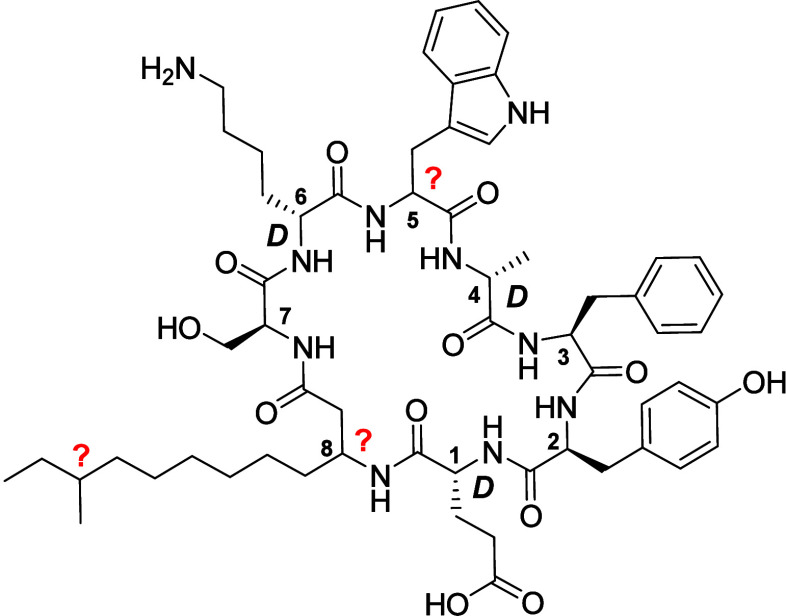
Recently proposed structure of paenilipoheptin A with stereochemical ambiguities indicated.^16^

While structurally distinct, the macrocyclic lipopeptide connectivity of the paenilipoheptins is reminiscent of the iturin class of lipopeptides produced by *B. subtilis*.^23,24^ As illustrated in Figure 1, paenilipoheptin A features a macrocyclic core composed of seven alpha amino acids, of which the residues at positions 2, 3, and 7 were previously shown to have l-stereochemistry while those at positions 1, 4, and 6 have d-stereochemistry (based on Marfey’s analysis).^15,16^ The macrocycle is closed by amide bond formation between the C-terminal d-Glu^1^ residue and the amino group of an unusual beta-amino acid bearing a lipophilic side chain containing anteiso-type branching. Notably, the experimental approaches underscoring the previously proposed structure of paenilipoheptin A were not sufficient to address the stereochemistry of the beta-amino acid at position 8 or to confirm the d-stereochemistry proposed for Trp^5^ (based on analysis of the biosynthetic gene cluster). To address these remaining stereochemical details, we therefore turned to a total synthesis approach.

To begin, we focused our attention on the unusual 13-carbon branched tail beta-amino acid at position 8. Given that this amino acid is not commercially available in any of the four possible stereochemical configurations, we opted to first synthesize a simpler, structurally similar, linear analog with the aim of gaining insight into the stereochemistry of the β-position. While methods have been reported for the synthesis of some beta-amino acids,^25,26^ these approaches were not deemed to be optimal for our purposes. Instead, we elected to employ an approach starting from the available and suitably protected l- and d-Asp building blocks **2a** and **2b**, using Wittig chemistry to install the lipid side chain (Scheme 1).^27^ Conversion of **2a** and **2b** to the corresponding aldehydes **3a** and **3b** was achieved as previously described.^28^ Wittig reagent **5** was prepared from the corresponding halide **4** via an established protocol.^29^ In our initial attempt at condensing **3a** with **5**, we employed a 2-fold excess of **5** treated with BuLi at −10 °C to generate the active ylide, followed by the addition of the aldehyde at −10 °C. While this approach failed to yield the desired product, we found that lowering the temperature to −78 °C during the aldehyde addition step led to the formation of product **6a** in 20% yield after column chromatography. Given this low yield, when preparing the enantiomeric **6b**, we explored the use of potassium bis(trimethylsilyl)amide (KHMDS) at room temperature to activate the ylide, followed by the addition of aldehyde **3b** at −78 °C. Gratifyingly, this approach was found to result in an improved 59% yield of the expected alkene product. With key intermediates **6a** and **6b** in hand, we proceeded with hydrogenation, which yielded the saturated **7a** and **7b** in quantitative yield. The Boc and tBu groups were then removed using TFA, followed by direct treatment with Fmoc-succinimide (FmocOSu) to yield Fmoc-protected **8a** and **8b** in 75% and 64% yields, respectively.

**Scheme 1:**
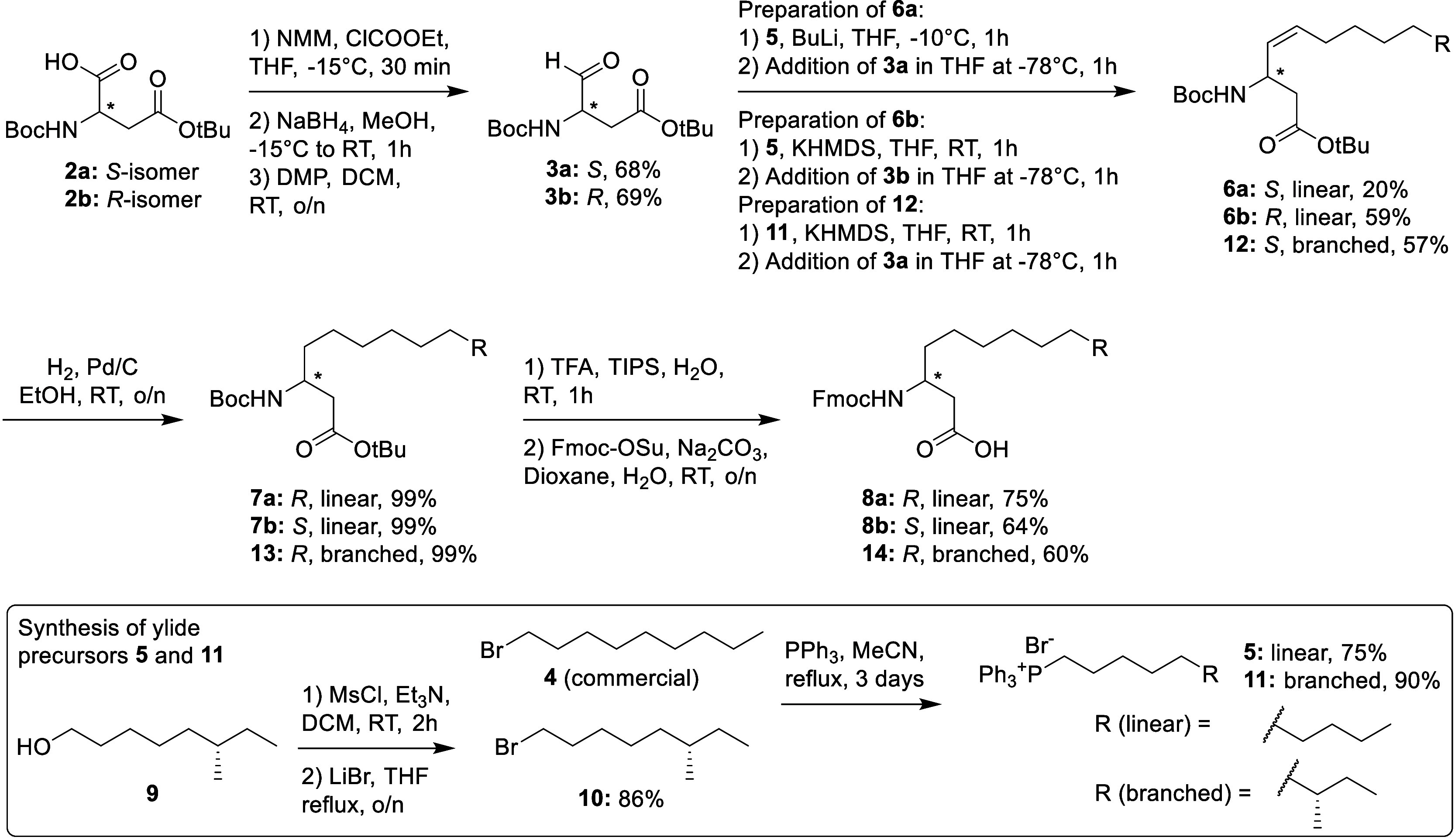
Syntheses of Beta-Amino Acids **8a**, **8b**, and **14**

With both enantiomers of the linear analogue of the beta-amino acid in hand, we proceeded to compare the retention times of their corresponding Marfey’s derivatives with that of the Marfey’s derivative obtained from hydrolysis of natural paenilipoheptin A (Table S1).^30,31^ To do so, we retained a portion of intermediates **7a** and **7b**, which, after removal of the Boc and tBu groups, were directly treated with Marfey’s reagent (1-fluoro-2-4-dinitrophenyl-5-l-alanine amide). Our reason for doing so was based on the hypothesis that the branched beta-amino acid derived from the natural product would have a similar retention time to the linear analogue bearing the same stereochemistry at the β-position. When comparing the retention times of the corresponding adducts, we found that Marfey’s derivative of amino acid **7a** elutes at nearly the same time as that derived from the natural product. On the other hand, the Marfey’s adduct derived from the enantiomeric **7b** had a significantly different retention time. These findings provided a hint that the β-position of the N-terminal beta-amino acid in paenilipoheptin A is of *R* stereochemistry. With regard to the unknown stereocenter in the anteiso-branched lipid side chain, we made the assumption that it is of the *S* configuration, as commonly seen in other compounds also bearing anteiso-branched lipids produced by *Paenibacillus* spp.^12,13,32^ Conveniently, (*S*)-6-methyloctan-1-ol (**9**) is commercially available, providing a short route to the corresponding Wittig reagent (Scheme 1). In doing so, alcohol **9** was first converted to the mesylate, followed by treatment with LiBr in THF to yield bromide **10**. To generate the phosphonium bromide **11**, we followed a similar method as used for the preparation of **5**, with purification achieved by precipitation from MTBE rather than column chromatography. With a limited supply of Wittig reagent **11**, we opted to use only 1.1 equiv of this compound in the subsequent Wittig reaction, otherwise following the same procedure used for the preparation of **6b**. The desired product **12** was thus formed in an acceptable 57% yield, after which hydrogenation yielded **13,** which, in turn, was subjected to the same deprotection/reprotection steps used for preparing **8a** and **8b**, to arrive at building block **14**.

With building block **14** in hand, we proceeded to synthesize paenilipoheptin A using a combined solid- and solution-phase strategy (Scheme 2). In doing so, we used microwave-assisted solid phase peptide synthesis (SPPS) to prepare the linear protected precursor that was subsequently cleaved from the resin and cyclized in the solution. Also of note, we specifically elected to install d-Trp at position 5 (as predicted based on analysis of the biosynthetic gene cluster; see Figure S1).^16^ As illustrated in Scheme 2, protected linear peptide **15** was synthesized starting from the d-Glu^1^ loaded via its α-carboxyl group onto 2-chlorotrityl (CT) resin. In preparing the linear precursor peptide 10% piperazine in NMP/EtOH was used for Fmoc deprotections and *N*,*N*,*N*′,*N*′-tetramethyl-*O*-(1*H*-benzotriazol-1-yl)uronium hexafluorophosphate (HBTU) and *N*,*N*-diisopropylethylamine (DIPEA) for amino acid couplings. Given its limited supply, the final coupling of compound **14** was performed manually using a reduced number of equivalents in comparison to the standard protocol used for the other couplings. After the final Fmoc deprotection, the protected peptide **15** was then cleaved from the resin using hexafluoroisopropanol (HFIP) to maintain all side-chain protecting groups. After evaporation, the crude was then directly treated with *N*,*N*′-diisopropylcarbodiimide (DIC)/ethyl cyanohydroxyiminoacetate (Oxyma) (6:6) in DCM/DMF (5:1), which resulted in the clean and complete formation of the desired macrocycle via amide bond formation between the amino group of the N-terminal beta-amino acid and the α-carboxyl group of d-Glu^1^. Following this, global deprotection was performed by treatment with TFA/TIPS/H_2_O, resulting in the desired **1b** as the main product as judged by LCMS. Interestingly, under these conditions, we did not achieve complete deprotection, with approximately 10% of the product observed as a + 44 amu species that we ascribed to the persistence of a CO_2_ adduct with the indole of d-Trp^5^. To resolve this issue, we further treated the crude product mixture with a 2% solution of acetic acid (AcOH) in H_2_O/MeCN at 40 °C for 30 min, which led to complete deprotection. Subsequent purification using reverse-phase high-performance liquid chromatography (RP-HPLC) resulted in a yield of 23 mg of compound **1b** (37% overall yield, corresponding to an average yield of 94.9% per step).

**Scheme 2:**
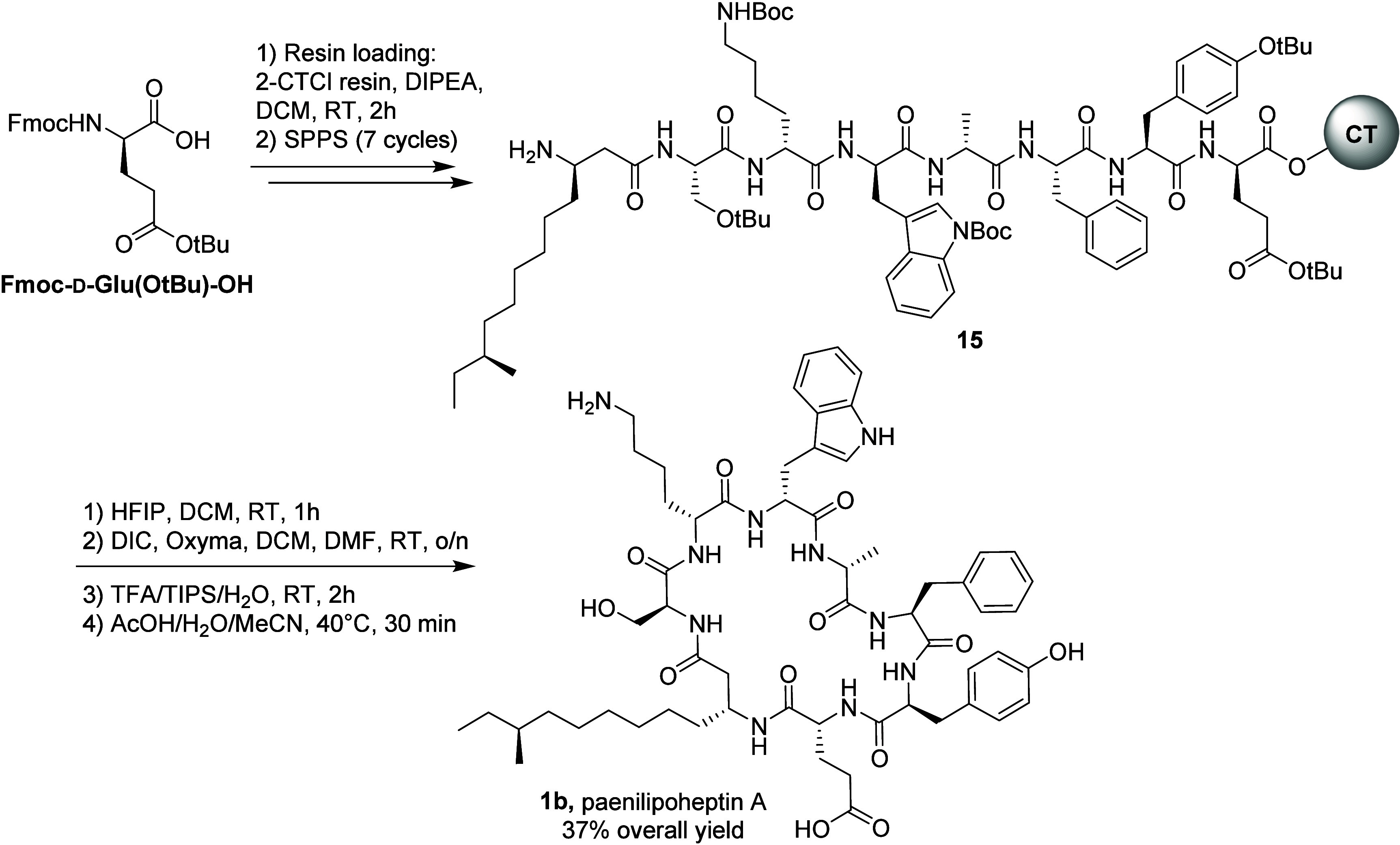
Combined Solid- and Solution-Phase Route Developed for the Synthesis of Compound **1b** (Paenilipoheptin A)

We next proceeded to compare compound **1b** with natural paenilipoheptin A isolated from its producing *Paenibacillus* strain. We were delighted to find that both compounds have identical NMR spectra (Figures S2 and S3) and show the same HPLC retention time, also eluting as a single peak when coinjected (Figure 2). These findings confirm that the structure of paenilipoheptin A is indeed that of compound **1b**.

**Figure 2:**
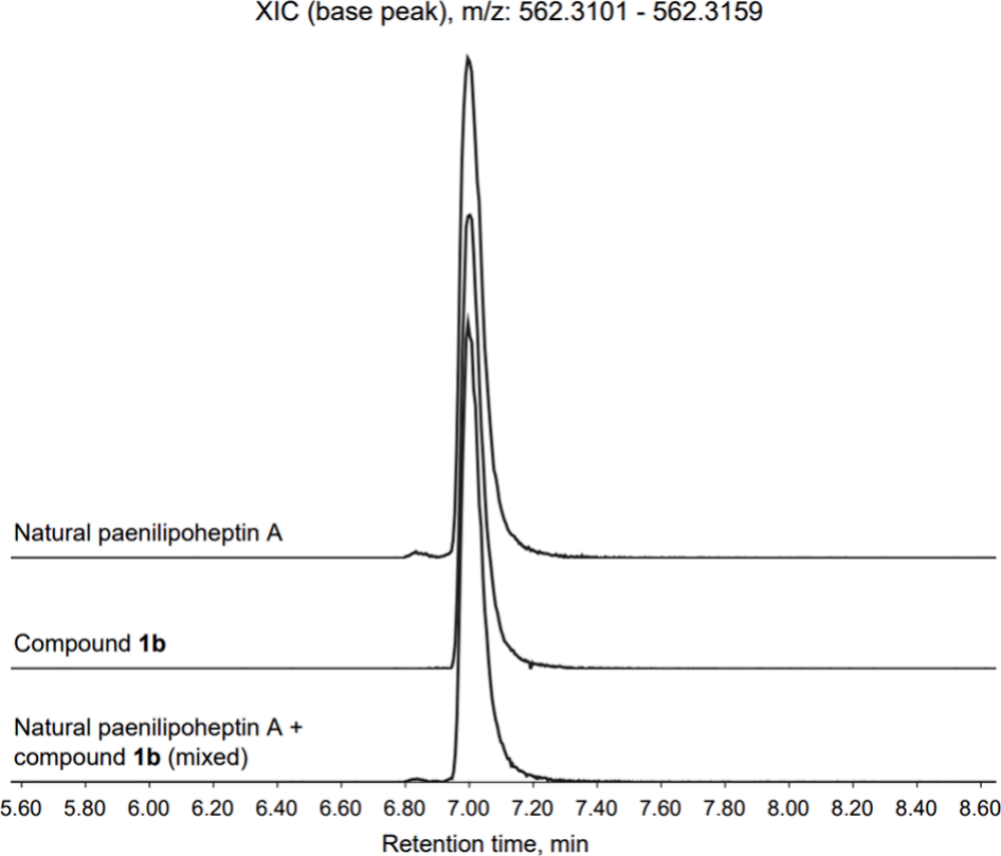
Extracted ion chromatograms of authentic paenilipoheptin A (*m*/*z* = 562.3130, calcd for [M+2H]^2+^) isolated from fermentation of *Paenibacillus* sp. JJ-21 overlaid with trace obtained for synthetic compound **1b**.

This further supports the stereochemistry of d-Trp^5^ and the 3*R* stereochemistry of the naturally occurring beta-amino acid. While these data also point to *S* stereochemistry for the anteiso-branched side chain, we cannot completely exclude the possibility of the natural product having the *R* configuration at this position.

In our previous work describing the isolation and partial characterization of paenilipoheptin A,^16^ it was not possible to perform a broad assessment of the compound’s bioactivity due to the limited amount of material isolated from the fermentation of the producing organism. Now, with access to larger quantities of material obtained via synthesis, we were able to perform a much wider screen against a range of bacteria and fungi (Table 1 and Table S2). Due to its limited aqueous solubility, we selected 64 μg/mL as the maximum concentration of compound **1b** used in the activity assays.

**Table 1:** MICs Determined for Naturally Produced Paenilipoheptin A and Compound **1b**

	MIC (μg/mL)		
Gram-positive bacterial strain tested	Paenilipoheptin A	**1b**	Vancomycin
*B. subtilis* 168	16	8	0.25
*E. faecium* E980	16	8	0.5
*E. faecium* VRE E155	ND^a^	8	>128
*E. faecium* VRE E7314	ND^a^	8	>128
*S. aureus* ATCC 29213	16	16	0.5
*S. aureus* USA 300	16	16	0.5
*S. aureus* VRS3b	ND^a^	16	>128
*S. aureus* LIM-2	ND^a^	32	4
*Paenibacillus* sp. JJ-21	ND^a^	64	0.25

a ND = not determined.

Interestingly, compound **1b** did not show any activity against the fungal strains tested, despite sharing some structural similarities with iturin A, a natural product reported to have antifungal activity.^23,33^ The extent to which the antifungal activity observed for iturin A is driven by a targeted mechanism or nonspecific membrane lysis can be debated, considering its propensity to lyse red blood cells.^34^ In contrast, when tested at the same concentration, **1b** exhibited no hemolytic activity (Figure S4).

When tested against a panel of bacteria, compound **1b** was found to display no activity against Gram-negative species, including a hypersensitive strain of *E. coli* with deletions of the *bamB* gene along with knockout of the *tolC* porin gene.^35^ However, when tested against Gram-positive species, **1b** was found to inhibit the growth of *B. subtilis* and *E. faecium* strains at a concentration of 8 μg/mL and also fully inhibited the growth of *S. aureus* at a concentration of 16 μg/mL, which is similar to the values obtained for paenilipoheptin A isolated from biological source. Interestingly, the same antibacterial activity was maintained against vancomycin-resistant strains of *S. aureus* and *E. faecium* with minimum inhibition concentrations (MICs) ranging from 8 to 16 μg/mL, while vancomycin showed no activity at the highest concentration tested of 128 μg/mL. We also tested the effect of compound **1b** on the growth of the Gram-positive paenilipoheptin A producing organism, *Paenibacillus sp*. JJ-21. In this case, a much higher MIC value (64 μg/mL) was measured, relative to the other Gram-positives tested, which may be indicative of a self-protective resistance mechanism used by the organism to shield itself from the effects of its own antibacterial compound. Finally, compound **1b** was also tested against *M. tuberculosis*, *M. smegmatis*, and *M. abscessus*, which, in all cases, indicated no impact on bacterial cell growth.

In conclusion, we report here the total synthesis and full structure elucidation of the natural product paenilipoheptin A. This represents the first total synthesis of a member of the paenilipoheptin family of lipopeptides, of which a number of members have recently been identified via bacterial genome mining. The synthesis of paenilipoheptin A depended on the preparation of a unique beta-amino acid containing (3*R*, 10*S*) stereochemistry. Synthetic paenilipoheptin A and the natural product isolated from the producing microorganism were shown to have identical analytical data supporting the fully assigned structure. Access to synthetic paenilipoheptin A enabled further assessment of its activity against a broad selection of bacterial and fungal strains revealing the compound to have specific anti-Gram-positive activity.

## Data Availability

The data underlying this study are available in the published article and its Supporting Information.

## Supporting Information Available

General information, synthetic procedures, Marfey’s analysis results, biosynthetic gene cluster analysis, comparison of ^1^H and ^13^C NMR data for natural and synthetic paenilipoheptin A, extended MIC table, hemolysis data, ^1^H NMR, ^13^C NMR, HRMS, and HPLC data for all key compounds (PDF)
