# Supplementary material for: Total Synthesis, Structure Elucidation, and Bioactivity Evaluation of the Cyclic Lipopeptide Natural Product Paenilipoheptin A
Source: Org Lett. 2025 Mar 18;27(12):2826–31. doi: 10.1021/acs.orglett.5c00232 (PMC11959586; doi:10.1021/acs.orglett.5c00232)
Supplement: Supplementary file 1 — ol5c00232_si_001.pdf [file ol5c00232_si_001.pdf]

Supporting Information

Total Synthesis, Structure Elucidation, and Bioactivity Evaluation of the Cyclic Lipopeptide Natural Product Paenilipoheptin A

Vladyslav Lysenko,[a,‡] Nataliia V. Machushynets,[b,‡] Jisca L. van Dam,[c] Fabienne A. C. Sterk,[a] Alexander Speer,[d] Arthur F. J. Ram,[c] Cornelis J. Slingerland,[a] Gilles P. van Wezel,[b,e,*] Nathaniel I. Martin[a,*]

[a] Biological Chemistry Group, Institute of Biology, Leiden University, 2333 BE, Leiden, The Netherlands

[b] Molecular Biotechnology Group, Institute of Biology, Leiden University, 2333 BE, Leiden, The Netherlands

[c] Fungal Genetics and Biotechnology Group, Institute of Biology, Leiden University, 2333 BE, Leiden, The Netherlands

[d] Department of Medical Microbiology and Infection Prevention, Amsterdam University Medical Centre, 1081 HZ, Amsterdam, The Netherlands

[e] Department of Microbial Ecology, Netherlands Institute of Ecology, 6700 PB, Wageningen, The Netherlands

‡ These authors contributed equally

* Corresponding authors: n.i.martin@biology.leidenuniv.nl, g.wezel@biology.leidenuniv.nl

Table of contents

	Page
**Table of contents**	2
**General information**	3-4
**Synthesis methods and analytical data on small molecules**	5-9
**Detailed procedures for solid-phase peptide synthesis of the compound 1b**	10-11
**Marfey`s analysis**	12
**Analysis of the paenilipoheptin A biosynthetic gene cluster**	13
**NMR comparison of natural paenilipoheptin A and compound 1b**	14-15
**Extended MIC table**	16
**Hemolysis**	17
**NMR Spectra**	18-28
**HPLC analysis of the peptide 1b**	29
**References**	30

General information

Reagents

All reagents employed were of American Chemical Society (ACS) grade or higher and were used without further purification unless otherwise stated.

HRMS

High-resolution mass spectra (HRMS) analyses were performed on a Shimadzu Nexera X2 UHPLC system with a Waters Acquity HSS C18 column (2.1 × 100 mm, 1.8 µm) at 30 °C and equipped with a diode array detector. The following solvent system, at a flow rate of 0.5 mL/min, was used: solvent A, 0.1 % formic acid in water; solvent B, 0.1 % formic acid in acetonitrile. Gradient elution was as follows: 95:5 (A/B) for 1 min, 95:5 to 15:85 (A/B) over 10 min, 15:85 to 0:100 (A/B) over 1 min, 0:100 (A/B) for 4 min, then reversion back to 95:5 (A/B) for 3 min. This system was connected to a Shimadzu 9030 QTOF mass spectrometer (ESI ionization) calibrated internally with Agilent's API-TOF reference mass solution kit (5.0 mM purine, 100.0 mM ammonium trifluoroacetate and 2.5 mM hexakis(1*H*,1*H*,3*H*-tetrafluoropropoxy)phosphazine) diluted to achieve a mass count of 10000. MZMine 3.2.8 was used to analyze the obtained data.[1]

Analytical HPLC

HPLC analyses were performed on a Shimadzu Prominence-i LC-2030 system with a Dr. Maisch ReproSil Gold 120 C18 column (4.6 × 250 mm, 5 µm) at 30 °C and equipped with a UV detector monitoring at 214 and 254 nm. The following solvent system, at a flow rate of 1 mL/min, was used: solvent A, 0.1 % TFA in water/acetonitrile 95/5; solvent B, 0.1 % TFA in water/acetonitrile 5/95. Gradient elution was as follows: 100:0 (A/B) for 3 min, 100:0 to 0:100 (A/B) over 47 min, 0:100 (A/B) for 4 min, then reversion back to 100:0 (A/B) over 1 min, 100:0 (A/B) for 5 min.

NMR

^{1}H and ^{13}C NMR spectra were recorded on Bruker AV 400 MHz (at 400 (^{1}H), 162 (^{31}P), and 101 (13C) MHz), AV 850 MHz (at 850 (1H), and 214 (^{13}C) MHz). The temperature of the NMR experiments was 298K unless stated otherwise. Chemical shifts are reported in ppm (δ) and were calibrated using residual deuterated solvent as an internal reference. (δ ^{1}H NMR: $CDCl_3$ 7.26; DMSO 2.50; δ ^{13}C NMR: $CDCl_3$ 77.16; DMSO 39.52). The NMR data are processed as follows: chemical shift, multiplicity (br s = broad singlet, s = singlet, d = doublet, dd = double doublet, t = triplet, dt = double triplet, q = quartet, tt = triple triplet, m = multiplet), integration, coupling constants (*J*, reported in Hz) and a number of nuclei. NMR spectra were analyzed and processed using MestreNova version 14.2.0. Structural assignments were made with additional information from NOESY, gCOSY, and gHSQC experiments.

Optical rotations

Optical rotations were measured on an automatic Anton Paar MCP100 polarimeter of sodium D-line, at λ = 589 nm.

Antibacterial assay against Gram-negative and Gram-positive bacteria

From glycerol stocks, bacterial strains were cultured on blood agar plates and incubated overnight at 37 °C. Following incubation, 3 mL of tryptic soy broth (TSB) was inoculated with an individual colony. The cultures were grown to exponential phase (OD_{600nm} = 0.5) at 37 °C. The bacterial suspensions were then diluted 100-fold in TSB (for *E. faecium* strains) or Mueller Hinton II Broth (MHIIB) to reach a bacterial cell density of 10^6 CFU mL^{-1}. In polypropylene 96-well microtiter plates, test compounds in assay media (e.g., MHIIB or TSB) were added in triplicate and two-fold serially diluted to achieve a final volume of 50 µL per well. An equal volume of bacterial suspension (50 µL, 10^6 CFU mL^{-1}) was added to the wells. The plates were sealed with breathable membranes and incubated at 37 °C for 18-22 h with constant shaking (600 rpm). The minimal inhibitory concentrations (MIC) were determined by visual inspection as the median of a minimum of triplicates.

Antibacterial assay against filamentous fungi and yeasts

The filamentous fungi strains were cultivated on malt extract agar (MEA) plates for 7 days, *Penicillium expansum* ATCC 24692 at 25 ˚C and *Aspergillus fumigatus* Af293, *Aspergillus niger* N400, *Aspergillus oryzae* RIB40 and *Fusarium oxysporum* CBS 101587 at 30 ˚C. Spores were harvested in sterile saline

solution (0.9% NaCl in demi water) and filtered. Spore concentrations were determined using a TC20 automated cell counter, and spore stocks were diluted to 10^6 spores/mL.

The yeast strains *Saccharomyces cerevisiae* NSY220.1 and *Candida albicans* ATCC 10231 were cultivated on yeast extract peptone dextrose agar (YPDA, 1% yeast extract, 2% bacteriological peptone, 2% glucose, 1.5% agar) for 5 days at 30 ˚C, followed by overnight liquid cultivations in 100 mL YPD (1% yeast extract, 2% bacteriological peptone, 2% glucose) at 30 ˚C, 200 rpm. Fresh cells were obtained by cultivation of 500 µL preculture in 100 mL fresh YPD at 30 ˚C, 200 rpm, until OD_{600nm} reached 0.4.

The MIC of the compounds on filamentous fungi and yeast strains was determined using a liquid assay utilizing 96-well micro test plates. Each well contains a total volume of 100 µL consisting of the tested compound in the growth medium, MEB for the filamentous fungi strains, and YPD for the yeast strains. For each compound, a concentration range was tested using a 2-fold dilution series. Compound **1b** and Iturin were tested in triplicate, Nystatin was tested in duplicate. Each well was inoculated with 10 µL of spore stock (10^4 spores) or cell suspension. Growth was scored after 7 days of incubation at the corresponding growth temperatures for each organism mentioned above. To limit evaporation and dehydration, the plates were kept closed and incubated in closed boxes. The MIC values were defined as the highest dilution, showing no growth.

Antibacterial assay against *M. tuberculosis* H37Rv, *M. smegmatis* mc^{2}155, *M. abscessus* ATCC 19977

The resazurin microtiter plate assay (REMA) was employed to assess the MIC of compounds against mycobacterial species. For this assay, the compound was prepared in a series of twofold dilutions within a 96-well plate using bacterial growth medium (7H9 Difco, 10% ADS, 0.2% glycerol, and 0.02% tyloxapol). Bacteria were grown until the mid-logarithmic phase, then collected by centrifugation, washed in PBS with 0.02% tyloxapol, resuspended in the growth medium, and introduced into the wells containing compound dilutions, aiming for a final OD_{600} of 0.001 per well. The plates were sealed with parafilm and incubated at 37°C for 1 day for *M. smegmatis*, 2 days for *Mycobacterium abscessus*, and 6 days for *M. tuberculosis*. After incubation, resazurin solution (0.025% [wt/vol] resazurin sodium salt in Milli-Q water with 20% Tween-80 in a 3:1 ratio) was added to each well, and plates were further incubated. Color change was observed to indicate the extent of bacterial growth, and fluorescence was measured using a BioTek plate reader (Synergy H1) with bottom reading mode, excitation at 560 nm, and emission at 590 nm. Data from each 96-well plate were normalized to wells treated with DMSO (considered 100% viability) following background subtraction (medium only). The MIC values were defined as the highest dilution, showing no growth.

Hemolysis assay

The hemolytic activity of compound **1b** was assessed in triplicate. Red blood cells from defibrillated sheep blood obtained from Thermo Fisher were centrifuged (400 g for 15 min at 4 °C) and washed with Phosphate-Buffered Saline (PBS) containing 0.002% Tween20 (buffer) five times. Then, the red blood cells were normalized to obtain a positive control read-out of 2.5 at 415 nm to stay within the linear range with the maximum sensitivity. A serial dilution of the compounds (64 to 2 µg/mL, 75 µL) was prepared in a 96-well plate. The outer border of the plate was filled with 75 µL buffer, the plate also contained a positive control (0.1% Triton-X final concentration, 75 µL) and a negative control (buffer, 75 µL) in triplicate. The normalized blood cells (75 µL) were added, and the plates were incubated at 37 °C for 1 h while shaking at 500 rpm. A flat-bottom plate of polystyrene with 100 µL buffer in each well was prepared. After incubation, the plate was centrifuged (800 g for 5 min at room temperature), and 25 µL of the supernatant was transferred to their respective wells in the flat-bottom plate. The values obtained from a read-out at 415 nm were corrected for background (negative control) and transformed to a percentage relative to the positive control (0.1% Triton-X).

Synthesis methods and analytical data on small molecules

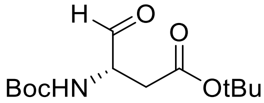

tert-butyl (*S*)-3-((tert-butoxycarbonyl)amino)-4-oxobutanoate (3a): (*S*)-4-tert-Butoxy-2-(tert-butoxycarbonylamino)-4-oxobutanoic acid (**2a**) (4 g, 13.83 mmol) was dissolved in anhydrous THF (50 mL) and the solution was cooled to -20°C while stirring under Ar atmosphere before the addition of *N*-methylmorpholine (1.52 mL, 13.83 mmol). After the reaction had been stirred for 10 min, ethyl chloroformate (1.32 mL, 13.83 mmol) was added dropwise, and the reaction was stirred for 1h. The sodium borohydride (1.57 g, 41.48 mmol) was then added, followed by dropwise addition of MeOH (5 mL) over 10 min at -20°C. After 1 hour, the reaction mixture was allowed to warm to room temperature and was left stirring for another hour before quenching with 1M HCl (50 mL). The organic solvents were separated and evaporated under reduced pressure, and the residue was combined with water fraction and extracted with MTBE (2 × 100 mL). The combined organic layer was washed with 1M HCl (70 mL), water (70 mL), sat. aqueous $NaHCO_3$ (70 mL) and brine (40 mL), then dried over Na_2SO_4 and evaporated under reduced pressure. The residue was dissolved in anhydrous DCM (80 mL), and Dess-Martin periodinane (6.45 g, 15.21 mmol) was added at room temperature to this solution. After 1h, the reaction was completed, as judged by TLC, the solvent was evaporated, and the residue was quenched with a 10% aqueous solution of $Na_2S_2O_3$ (70 mL) and extracted with MTBE (2 × 100 mL). The combined organic layer was washed with a saturated aqueous solution of $NaHCO_3$ (80 mL), water (80 mL), and brine (40 mL), dried over Na_2SO_4, filtered, and evaporated to give the crude product that was purified using column chromatography (MTBE/petroleum ether (PE) = 1:2, R_f = 0.3) which yielded the pure aldehyde **3a** (2.52 g, 68% yield) as a yellow semi-solid.

^{1}H NMR (400 MHz, $CDCl_3$) δ 9.61 (s, 1H), 5.62 (d, *J* = 7.7 Hz, 1H), 4.34 – 4.25 (m, 1H), 2.87 (dd, *J* = 17.0, 4.8 Hz, 1H), 2.71 (dd, *J* = 17.0, 5.0 Hz, 1H), 1.43 (s, 9H), 1.41 (s, 9H).

^{13}C{^{1}H} NMR (101 MHz, $CDCl_3$) δ 199.6, 170.4, 155.6, 82.1, 80.5, 56.3, 35.9, 28.4, 28.1.

Spectral data is in agreement with those reported in the literature.[2]

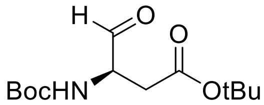

tert-butyl (*R*)-3-((tert-butoxycarbonyl)amino)-4-oxobutanoate (3b): The compound was prepared with a similar procedure to that employed for the synthesis of compound **3a** starting from tert-butyl (*R*)-3-((tert-butoxycarbonyl)amino)-4-hydroxybutanoate (**2b**) (4 g, 13.83 mmol). Yield: 2.6 g, 69%, yellow semi-solid. Spectral data is consistent with the structure and identical to *S*-isomer.

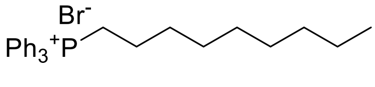

Nonyltriphenylphosphonium Bromide (5): To a stirred solution containing triphenylphosphine (4.11 g, 15.7 mmol) in 80 mL of anhydrous MeCN was added 1-bromononane (3 mL, 15.7 mmol) at room temperature. The reaction mixture was stirred at reflux for 3 days to achieve full conversion of starting materials as judged by ^{1}H-NMR. The cooled reaction mixture was concentrated and purified via column chromatography (DCM/MeOH = 10:1, R_f = 0.4) to afford compound **5** (5.52 g, 75% yield) as a white solid. Prior to use in the next step, the Wittig reagent was co-evaporated with anhydrous THF.

^{1}H NMR (400 MHz, $CDCl_3$) δ 7.70 – 7.60 (m, 9H), 7.60 – 7.51 (m, 6H), 3.49 – 3.37 (m, 2H), 1.52 – 1.37 (m, 4H), 1.14 – 0.95 (m, 10H), 0.65 (t, *J* = 6.9 Hz, 3H).

^{13}C{^{1}H} NMR (101 MHz, $CDCl_3$) δ 134.84, 134.81, 133.3, 133.2, 130.3, 130.2, 118.2, 117.4, 31.4, 30.12, 30.0, 28.8, 22.6, 22.23, 22.20, 22.1, 13.8.

^{31}P{^{1}H} NMR (162 MHz, $CDCl_3$) δ 24.5.

Spectral data is in agreement with those reported in the literature.[3]

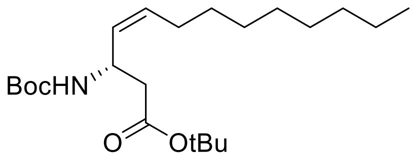

tert-butyl (*S*,*Z*)-3-((tert-butoxycarbonyl)amino)tridec-4-enoate (6a): BuLi (4 mL, 2.5M in Hexane, 10.00 mmol) was added dropwise to a stirred solution of Wittig reagent **5** (5.03 g, 10.71 mmol) in anhydrous THF (25 mL) at -15°C under Ar atmosphere. After 1 h of stirring at this temperature, the reaction was cooled to -78°C, and the aldehyde **3a** (1.3 g, 4.76 mmol) in anhydrous THF (5 mL) was added dropwise. The reaction was allowed to warm up to room temperature and stirred for an additional hour. The reaction mixture was quenched with saturated aqueous NH_4Cl (25 mL) and extracted with Et_2O (2 × 50 mL). The combined organic phases were washed with 1M HCl (50 mL), sat. $NaHCO_3$ (50 mL) and brine (30 mL), then dried over Na_2SO_4, filtered, and concentrated in vacuo. Purification by column chromatography (MTBE/PE = 1:8, R_f = 0.42) afforded compound **6a** (0.36 g, 20% yield) as a mixture of diastereomers with the ratio 91:9 based on ^{1}H-NMR integration. NMR is reported for major isomer.

^{1}H NMR (400 MHz, $CDCl_3$) δ 5.49 – 5.41 (m, 1H), 5.37 – 5.29 (m, 1H), 5.05 (s, 1H), 4.75 – 4.63 (m, 1H), 2.45 (d, *J* = 5.7 Hz, 2H), 2.22 – 2.02 (m, 2H), 1.42 (d, *J* = 4.1 Hz, 18H), 1.37 – 1.19 (m, 12H), 0.86 (t, 3H).

^{13}C{^{1}H} NMR (101 MHz, $CDCl_3$) δ 170.7, 155.0, 133.1, 128.9, 81.0, 45.2, 41.6, 32.0, 29.7, 29.6, 29.42, 29.41, 28.5, 28.2, 27.8, 22.8, 14.2.

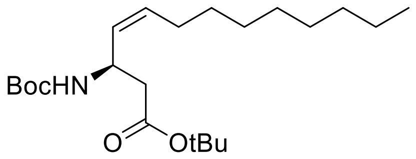

tert-butyl (*R*,*Z*)-3-((tert-butoxycarbonyl)amino)tridec-4-enoate (6b): KHMDS (20.3 mL, 0.5M in Toluene, 10.14 mmol) was added dropwise to a stirred solution of Wittig reagent **5** (5.1 g, 10.87 mmol) in anhydrous THF (25 mL) at room temperature under Ar atmosphere. After 1 h, the reaction was cooled to -78°C, and the aldehyde **3b** (1.32 g, 4.83 mmol) in anhydrous THF (5 mL) was added dropwise. The reaction was allowed to warm up to room temperature and stirred for an additional hour. The reaction mixture was quenched with saturated aqueous NH_4Cl (25 mL) and extracted with Et_2O (2 × 50 mL). The combined organic phases were washed with 1M HCl (50 mL), sat. $NaHCO_3$ (50 mL) and brine (30 mL), then dried over Na_2SO_4, filtered, and concentrated in vacuo. Purification by column chromatography (MTBE/PE = 1:8, R_f = 0.42) afforded compound **6b** (1.09 g, 59% yield) as a mixture of diastereomers with the ratio 91:9 based on ^{1}H-NMR integration. Spectral data is consistent with the structure and identical to *S*-isomer.

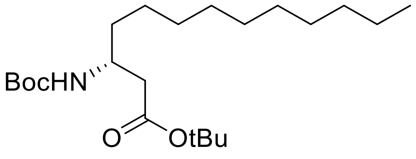

tert-butyl (*R*)-3-((tert-butoxycarbonyl)amino)tridecanoate (7a): A mixture of alkene **6a** (360 mg, 0.94 mmol) and Pd/C (50 mg, 10% Pd on charcoal) in EtOH (5 mL) was stirred under an atmosphere of H_2 baloon (1 atm) overnight. After the completion of the reaction, the catalyst was filtered off, the precipitate was washed with EtOH (3 × 10 mL), and the solution was concentrated in vacuo to give compound **7a** (360 mg, 99%) as a colorless oil.

^{1}H NMR (400 MHz, $CDCl_3$) δ 4.91 (d, *J* = 9.3 Hz, 1H), 3.91 – 3.78 (m, 1H), 2.46 – 2.31 (m, 2H), 1.47 – 1.39 (m, 20H), 1.34 – 1.20 (m, 16H), 0.87 (t, *J* = 6.7 Hz, 3H).

^{13}C{^{1}H} NMR (101 MHz, $CDCl_3$) δ 171.3, 155.5, 80.9, 48.0, 40.7, 35.0, 32.0, 29.73, 29.68, 29.54, 29.46, 28.5, 28.2, 26.2, 22.8, 14.2.

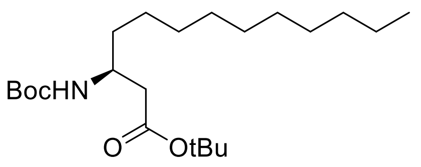

tert-butyl (*S*)-3-((tert-butoxycarbonyl)amino)tridecanoate (7b): The compound was prepared with a similar procedure to that employed for the synthesis of compound **7a** starting from alkene **6b** (1.05 g, 2.74 mmol). Yield: 1.05 g, 99%, colorless oil. Spectral data is consistent with the structure and identical to *R*-isomer.

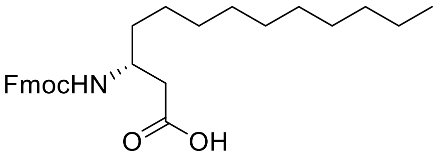

(*R*)-3-((((9H-fluoren-9-yl)methoxy)carbonyl)amino)tridecanoic acid (8a): The compound **7a** (360 mg, 0.93 mmol) was dissolved in a mixture of TFA/TIPS/H_2O (95:2.5:2.5, 3 mL) and was left stirring for 1h. Then, the reaction mixture was concentrated in vacuo, and the residue was dissolved in a mixture of water/Dioxane (1:1, 10 mL). Na_2CO_3 (297 mg, 2.80 mmol) was added to this suspension, and the resulting mixture was cooled to 0°C, followed by a dropwise addition of a solution of Fmoc-OSu (347 mg, 1.03 mmol) in dioxane (2 mL). The reaction mixture was stirred at room temperature overnight and next day diluted with water (20 mL) and extracted with Et_2O (25 mL). The aqueous layer was separated, acidified with 1M HCl till pH 2-3, and extracted with EtOAc (2 × 20 mL). The combined organic layers were washed with water (20 mL), brine (15 mL), dried over Na_2SO_4, filtered, and concentrated in vacuo. The residue was triturated with PE (5 mL), and the precipitate was filtered, washed with additional PE (2 × 5 mL), and dried to obtain Fmoc amino acid **8a** (320 mg, 76%) as a white powder.

$[\alpha]_D^{25}$ = +13.1° (c = 1, $CHCl_3$)

^{1}H NMR (400 MHz, DMSO-*d$_6$*) δ 12.15 (br s, 1H), 7.89 (d, *J* = 7.5 Hz, 2H), 7.68 (dd, *J* = 7.6, 3.8 Hz, 2H), 7.41 (t, *J* = 7.4 Hz, 2H), 7.31 (td, *J* = 7.4, 3.3 Hz, 2H), 7.21 (d, *J* = 8.7 Hz, 1H), 4.34 – 4.16 (m, 3H), 3.82 – 3.70 (m, 1H), 2.41 – 2.20 (m, 2H), 1.44 – 1.33 (m, 2H), 1.28 – 1.14 (m, 16H), 0.82 (t, *J* = 6.6 Hz, 3H).

^{13}C{^{1}H} NMR (101 MHz, DMSO-*d$_6$*) δ 172.6, 155.6, 144.0, 143.9, 140.8, 127.6, 127.0, 125.2, 120.1, 65.1, 47.8, 46.8, 34.2, 31.3, 29.04, 28.99, 28.8, 28.7, 25.3, 22.1, 14.0.

HRMS (ESI) m/z: $[M+H]^+$ calcd for $C_{28}H_{38}NO_4+H^+$: 452.2795; found: 452.2799.

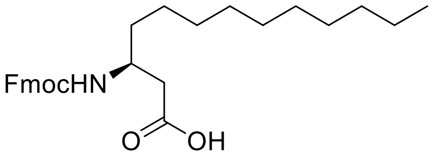

(*S*)-3-((((9H-fluoren-9-yl)methoxy)carbonyl)amino)tridecanoic acid (8b): The compound was prepared with a similar procedure to that employed for the synthesis of compound **8a** starting from compound **7b** (1.02 g, 2.645 mmol). Yield: 760 mg, 64% white powder. **$[\alpha]_D^{25}$** = -11.6° (c = 1, $CHCl_3$). **HRMS** (ESI) m/z: $[M+H]^+$ calcd for $C_{28}H_{38}NO_4+H^+$: 452.2795; found: 452.2820. Spectral data are consistent with the structure and identical to *R*-isomer.

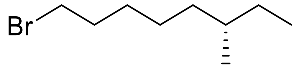

(*S*)-1-bromo-6-methyloctane (10): MsCl (350 mg, 3.05 mmol) was added dropwise to the solution of (*S*)-6-methyl octanol (400 mg, 2.77 mmol) and Et_3N (970 mkl, 6.94 mmol) in anhydrous DCM (10mL) at 5°C under Ar atmosphere. After 2 h, the solvent was evaporated, and the residue was diluted with water (10 mL) and extracted with Et_2O (40 mL). The organic layer was separated and washed with 1M HCl (10 mL), sat. aq. $NaHCO_3$ (10 mL), water (10 mL), brine (10 mL), then dried over Na_2SO_4, filtered, and concentrated in vacuo to afford corresponding mesylate that was then dissolved in THF (10 mL). LiBr (962 mg, 11.08 mmol) was added to this solution, and the reaction was refluxed overnight. The next day, the solvent was evaporated, and the residue was diluted with water (10 mL) and extracted with Et_2O (25

mL). The organic layer was washed with sat. aq. $NaHCO_3$ (10 mL), water (10 mL), brine (10 mL), then dried over Na_2SO_4, filtered, and concentrated in vacuo to afford bromide **10** (493 mg, 86% yield) as a colorless liquid.

^{1}H NMR (400 MHz, $CDCl_3$) δ 3.41 (t, *J* = 6.9 Hz, 2H), 1.86 (p, *J* = 7.0 Hz, 2H), 1.47 – 1.21 (m, 7H), 1.20 – 1.02 (m, 2H), 0.90 – 0.81 (m, 6H).

^{13}C{^{1}H} NMR (101 MHz, $CDCl_3$) δ 36.5, 34.4, 34.2, 33.0, 29.6, 28.7, 26.4, 19.3, 11.5.

Spectral data is in agreement with those reported in the literature.[4]

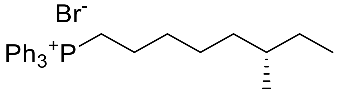

(*S*)-6-methyloctyltriphenylphosphonium bromide (11): To a stirred solution containing triphenylphosphine (625 mg, 2.38 mmol) in 20 mL of anhydrous MeCN was added bromide **10** (493 mg, 2.38 mmol) at room temperature. The reaction mixture was stirred at reflux for 3 days to achieve full conversion of starting materials as judged by NMR. The cooled reaction mixture was concentrated in vacuo, dissolved in a minimum amount of MeCN (2 mL), and crushed out from MTBE (20 mL). The liquid layer was decanted, and the precipitate was dried to afford compound **11** (1g, 90% yield) as a white solid.

^{1}H NMR (400 MHz, $CDCl_3$) δ 7.90 – 7.75 (m, 12H), 7.73 – 7.63 (m, 6H), 3.85 – 3.74 (m, 2H), 1.61 (d, *J* = 5.6 Hz, 4H), 1.31 – 1.12 (m, 5H), 1.10 – 0.93 (m, 2H), 0.83 – 0.73 (m, 6H).

^{13}C{^{1}H} NMR (101 MHz, $CDCl_3$) δ 135.09, 135.06, 133.9, 133.8, 130.7, 130.5, 119.0, 118.2, 36.2, 34.4, 30.9, 30.8, 29.5, 26.9, 23.2, 22.83, 22.79, 22.65, 19.3, 11.5.

^{31}P{^{1}H} NMR (162 MHz, $CDCl_3$) δ 25.0.

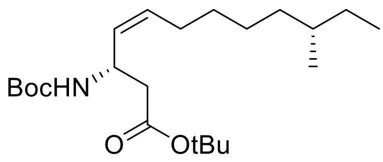

tert-butyl (3*S*,10*S*,*Z*)-3-((tert-butoxycarbonyl)amino)-10-methyldodec-4-enoate (12): KHMDS (1.83 mL, 0.5M in Toluene, 0.915 mmol) was added dropwise to a stirred solution of Wittig reagent **11** (473 mg, 1.01 mmol) in anhydrous THF (10 mL) at room temperature under Ar atmosphere. After 1 h, the reaction was cooled to -78°C, and the aldehyde **3a** (250 mg, 0.915 mmol) in anhydrous THF (2 mL) was added dropwise. The reaction was allowed to warm up to room temperature over 1 h and stirred for an additional hour. The reaction mixture was then quenched with saturated aqueous NH_4Cl (15 mL) and extracted with Et_2O (2 × 30 mL). The combined organic phases were washed with 1M HCl (30 mL), sat. $NaHCO_3$ (30 mL), and brine (10 mL), then dried over Na_2SO_4, filtered, and concentrated in vacuo. Purification by column chromatography (MTBE/PE = 1:8, Rf = 0.43) afforded compound **12** (200 mg, 57% yield) as a mixture of diastereomers with the ratio 92:8. NMR is reported for major isomer.

^{1}H NMR (400 MHz, $CDCl_3$) δ 5.48 – 5.36 (m, 1H), 5.36 – 5.25 (m, 1H), 5.16 – 4.95 (m, 1H), 4.66 (p, *J* = 6.6 Hz, 1H), 2.42 (d, *J* = 5.7 Hz, 2H), 2.19 – 2.00 (m, 2H), 1.46 – 1.35 (m, 18H), 1.34 – 1.17 (m, 7H), 1.13 – 0.99 (m, 2H), 0.85 – 0.77 (m, 6H).

^{13}C{^{1}H} NMR (101 MHz, $CDCl_3$) δ 170.7, 155.0, 133.0, 132.0, 128.8, 80.9, 45.2, 41.5, 36.6, 34.4, 30.0, 29.5, 28.5, 28.1, 27.8, 26.8, 19.3, 11.5.

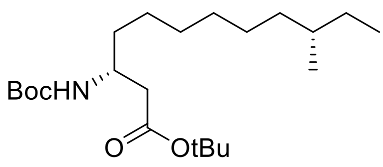

tert-butyl (3*R*,10*S*)-3-((tert-butoxycarbonyl)amino)-10-methyldodecanoate (13): The compound was prepared with a similar procedure to that employed for the synthesis of compound **7a** starting from alkene **12** (200 mg, 0.52 mmol). Yield: 200 mg, 99%, colorless oil.

^{1}H NMR (400 MHz, $CDCl_3$) δ 4.92 (d, *J* = 9.4 Hz, 1H), 3.92 – 3.79 (m, 1H), 2.45 – 2.30 (m, 2H), 1.48 – 1.37 (m, 20H), 1.35 – 1.17 (m, 11H), 1.16 – 1.01 (m, 2H), 0.88 – 0.78 (m, 6H).

^{13}C{^{1}H} NMR (101 MHz, $CDCl_3$) δ 171.2, 155.5, 80.9, 79.1, 47.9, 40.7, 36.7, 35.0, 34.5, 30.0, 29.59, 29.56, 28.5, 28.2, 27.1, 26.2, 19.3, 11.5.

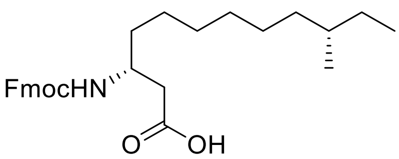

(3*R*,10*S*)-3-((((9H-fluoren-9-yl)methoxy)carbonyl)amino)-10-methyldodecanoic acid (14): The compound was prepared with a similar procedure to that employed for the synthesis of compound **8a** starting from compound **13** (200 mg, 0.52 mmol). Yield: 140 mg, 60%.

$[\alpha]_D^{25}$ = +12.7° (c = 1, $CHCl_3$).

^{1}H NMR (400 MHz, $CDCl_3$) δ 9.37 (br s, 1H), 7.75 (d, *J* = 7.6 Hz, 2H), 7.59 (d, *J* = 7.4 Hz, 2H), 7.39 (t, *J* = 7.5 Hz, 2H), 7.31 (t, *J* = 7.4 Hz, 2H), 5.90 (d, *J* = 8.9 Hz, 0.25H, minor rotamer), 5.19 (d, *J* = 9.1 Hz, 0.75H, major rotamer), 4.65 – 4.34 (m, 2H), 4.22 (t, *J* = 6.9 Hz, 1H), 4.05 – 3.92 (m, 0.75H, major rotamer), 3.81 – 3.69 (m, 0.25H, minor rotamer), 2.69 – 2.51 (m, 1.5H, major rotamer), 2.45 – 2.28 (m, 0.5H, minor rotamer), 1.62 – 1.17 (m, 13H), 1.17 – 1.03 (m, 2H), 0.89 – 0.81 (m, 6H).

^{13}C{^{1}H} NMR (101 MHz, $CDCl_3$) δ 177.1, 156.1, 144.1, 144.0, 141.4, 127.8, 127.2, 125.21, 125.17, 120.1, 67.5, 66.8, 48.9, 48.2, 47.4, 39.0, 36.7, 34.5, 30.00, 29.6, 29.5, 27.1, 26.3, 19.3, 11.5.

HRMS (ESI) m/z: $[M+H]^+$ calcd for $C_{28}H_{38}NO_4+H^+$: 452.2795; found: 452.2820.

<u>Detailed procedures for solid-phase peptide synthesis of the compound 1b</u>

Resin loading

2-Chlorotrityl chloride resin (2-CTC) (4 g, 1.51 mmol/g) was loaded by coupling via the free carboxyl group of Fmoc-D-Glu(OtBu)-OH (5.14 g, 12.08 mmol, 2 eq.) with DIPEA (5.26 mL, 30.2 mmol, 5 eq.) in 70 mL of DCM. After 3h at room temperature, an extra amount of DIPEA (5.26 mL, 30.2 mmol, 5 eq.) and 24 mol of MeOH were added, and the reaction was left shaking for another 15 minutes. The resin was then filtered, washed with DMF, EtOH, DCM, Et_2O, and dried overnight under a stream of N_2. The resin loading was then determined to be 0.555 mmol/g.

Automated solid-phase peptide synthesis

A CEM Liberty Blue automated peptide synthesizer with microwave irradiation was used to perform solid-phase peptide synthesis (SPPS). Synthesis was performed on a 0.05 mmol scale using the next reagents system: 0.05 mmol scale – 5eq. HBTU (0.25M in DMF), 10eq. DIPEA (0.5M in DMF), 5eq. of amino acid (0.2M in DMF). Fmoc group removal was performed using Piperazine/EtOH/NMP (1:1:9, m/v/v). A detailed overview of the automated protocols can be found below.

Resin swelling

The resin was swollen in 10 ml of DMF for 300 seconds prior to the first coupling.

Protocol 1: Standard Coupling protocol

Step	Function	Duration/Temperature
**1**	Deprotection N-terminus	50 s at 25°C then 250 s at 50°C
**2**	Wash (DMF)	RT
**3**	Wash (DMF)	RT
**4**	Wash (DMF)	RT
**5**	Coupling amino acid	150 s at 25°C then 750 s at 50°C

Protocol 2: Final deprotection N-terminus

Step	Function	Duration/Temperature
**1**	Deprotection N-terminus	50 s at 25°C then 250 s at 50°C
**2**	Wash (DMF)	RT
**3**	Wash (DMF)	RT
**4**	Wash (DMF)	RT

Procedure for the synthesis of the compound 1b

2-CT resin loaded with Fmoc-D-Glu(tBu)-OH was transferred into a CEM Liberty Blue µwave peptide synthesizer at a 0.05 mmol scale. The amino acids were coupled using standard coupling protocol (protocol 1) in the following order: 1) Fmoc-Tyr(tBu)-OH 2) Fmoc-Phe-OH; 3) Fmoc-D-Ala-OH; 4) Fmoc-D-Trp(Boc)-OH; 5) Fmoc-D-Lys(Boc)-OH; 6) Fmoc-Ser(tBu)-OH followed by a final deprotection using protocol 2. The resin was then transferred to a manual reactor for the solid phase peptide synthesis (SPPS) connected to nitrogen flow to perform the last coupling of the unnatural amino acid X. After the resin was swollen in DMF (7 ml) for 1 min, protected amino acid **12** (45.16 mg, 0.1 mmol), HBTU (37.92 mg, 0.1 mmol) and DIPEA (35.6 mkl, 0.2 mmol) were added. After bubbling with N_2 for 3h, the resin was filtered, washed with DMF (2 × 7 ml), and then treated with 7ml of Piperazine/EtOH/NMP (1:1:9, m/v/v) for 20 min, followed by washing with DMF (2 × 7 ml), DCM (2 × 7 ml). The peptide was then detached from the resin using 7 ml of the 20% hexafluoroisopropanol (HFIP) in DCM and filtered. Filtrate was

collected, and the solvents were removed by rotary evaporation, yielding the protected linear peptide, which was used directly in the next step.

Dry DMF (5 ml) was poured into the round-bottom flask containing a stirring bar, protected peptide (0.5 mmol), and Oxyma (43 mg, 0.3 mmol), followed by the addition of dry DCM (55 ml). The DIC (47 μL, 0.3 mmol) was added to the resulting mixture, and the reaction was left stirring overnight under an Argon atmosphere. The next day, the solvents were evaporated under reduced pressure, and the residue was diluted with water (20 ml) and extracted with EtOAc (2 × 30 ml). The combined organic layers were washed with 1M $NaHCO_3$ (25 ml), water (25 ml), brine (20 ml), dried under Na_2SO_4, and concentrated using rotary evaporation. Final sidechain deprotection was carried out by treating the obtained powder with 3 ml of the TFA/TIPS/H_2O (95:2.5:2.5) mixture for 2.5 h. The reaction mixture was precipitated in MTBE/Petroleum ether (1:1) and centrifuged (4500 rpm, 5 min). The pellet was then resuspended in MTBE/Petroleum ether (1:1) and centrifuged again (4500 rpm, 5 min). Finally, the pellet containing the crude peptide was dissolved in 10 ml of 2% AcOH in the H_2O/MeCN (70:30) and left at 40°C for 40 min, after which the solution was left to cool down to room temperature.

Peptide was purified via preparative HPLC using a BESTA-Technik system with a Dr. Maisch Reprosil Gold 120 C18 column (25 × 250 mm, 10 µm) and equipped with an ECOM Flash UV detector monitoring at 214 nm. The following solvent system, at a flow rate of 12 mL/min, was used: solvent A, 0.1 % TFA in water/acetonitrile 95:5; solvent B, 0.1 % TFA in water/acetonitrile 5:95. Gradient elution was as follows: 70:30 (A/B) for 3 min, 70:30 to 0:100 (A/B) over 49 min, 0:100 (A/B) for 4 min, then reversion back to 70:30 (A/B) over 1 min, 70:30 (A/B) for 3 min. The fractions contained the product were combined and lyophilized to obtain compound **1b** as a white fluffy powder (23 mg, 37% yield, calcd for mono TFA salt) with >95% purity as determined by HPLC.

HRMS (ESI) m/z: $[M+2H]^{2+}/2$ calcd for $C_{59}H_{82}N_{10}O_{12}+2H^+$: 562.3130; found: 562.3134.

Marfey`s analysis

The stereochemistries of chiral centers present at α carbons were assigned by applying derivatization methods coupled with chromatographic analysis. The advanced Marfey's method using L-FDAA (1-fluoro-2-4-dinitrophenyl-5-L-alanine amide) established the absolute configurations of amino acids.[5]

The general method for Marfey`s analysis (as described):[6]

A sample of authentic paenilipoheptin A (30 µg) in 6M HCl (100 µL) was heated to 100 °C in a sealed vial for 15 h, after which the hydrolysate was concentrated to dryness at 40 °C under a stream of dry N_2. The hydrolysate was then treated with 1 M $NaHCO_3$ (20 µL) and L-FDAA (1% solution in acetone, 40 µL) at 40 °C for 1 h, after which the reaction was neutralized with 1 M HCl (20 µL). An aliquot of the analyte was diluted 50 times with H_2O/MeCN (1:1) and injected (2 µL) into an HRMS instrument following the standard protocol of the analysis (see general methods). The analyte amino acid content was assessed by comparison to authentic standards, which were prepared from **7a**, **7b**, and **13** by treating the compounds with pure TFA to perform Boc and tBu deprotection. After that, all volatile components were evaporated in vacuo, and the residue was treated with solutions of $NaHCO_3$ and L-FDAA (or D-FDAA, see **Table S1**), as described above.

Table S1. Retention times (t_R, min) of the FDAA derivatives for the β-fatty amino acid (FAA) derived from authentic paenilipoheptin and compounds **7a**, **7b**, **13**. Marfey`s adduct made from compound **13** showed the same retention time as the one derived from the natural product.

	[M+H]+	t_R, min				
		**7a** (L-FDAA)	**7b** (L-FDAA)	**13** (L-FDAA)	**13** (D-FDAA)	Paenilipoheptin A (L-FDAA)
**FAA**	482.2609	10.35	9.70	10.17	9.53	10.17

Analysis of the paenilipoheptin A biosynthetic gene cluster

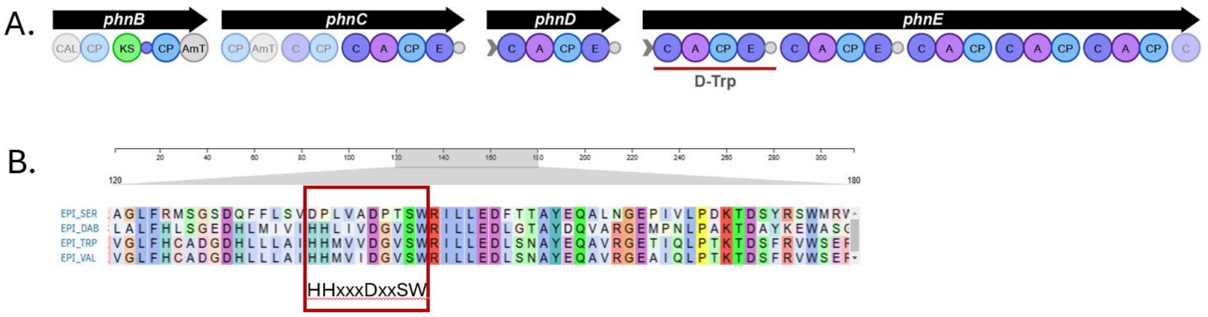

Figure S1. A. Module architecture of NRPS enzymes involved in the biosynthesis of paenilipoheptin A. Note that module with predicted substrate specificity of A domains for Trp contains epimerization (E) domain, indicating D-configuration. B. Clustal2 multiple sequence alignments of the epimerization domains of the paenilipoheptin A BGC from *Paenibacillus* sp. JJ-21. This revealed that the conserved active site motif HHxxxD of Trp is intact. Therefore, we believe that the epimerization (E) domain detected in this module is functional.

NMR comparison of natural paenilipoheptin A and compound 1b

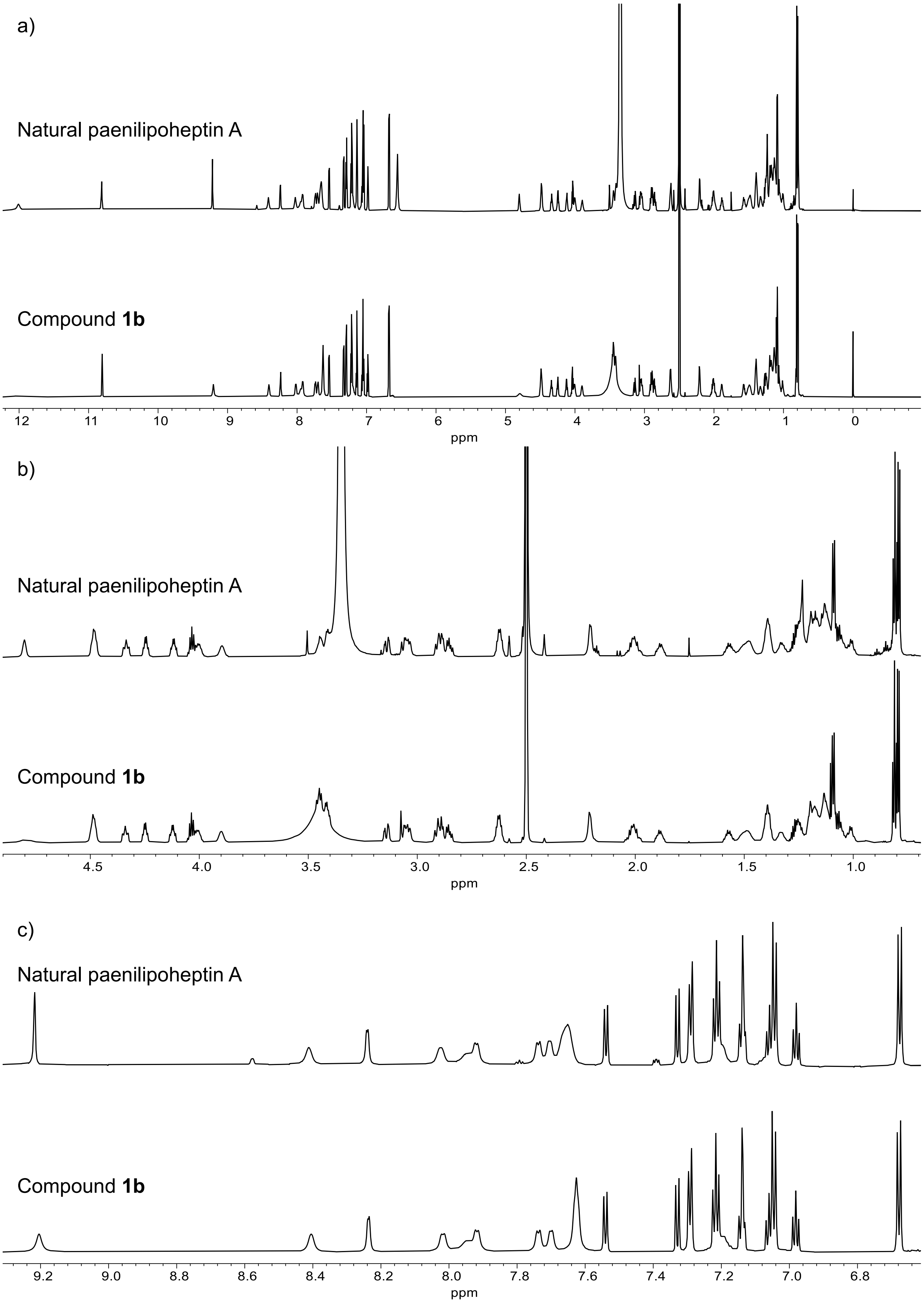

Figure S2. NMR comparison of the ^{1}H-NMR (850 MHz, DMSO-d_6) spectrum of natural paenilipoheptin A isolated after fermentation of the producing organism overlaid with ^{1}H-NMR (850 MHz, DMSO-d_6) spectra of synthetic compound **1b**: a) full spectrum, 12.5 – -1 ppm region; b) aliphatic protons region, 4.9 – 0.7 ppm; c) aromatic/amide protons region, 9.3 – 6.6 ppm.

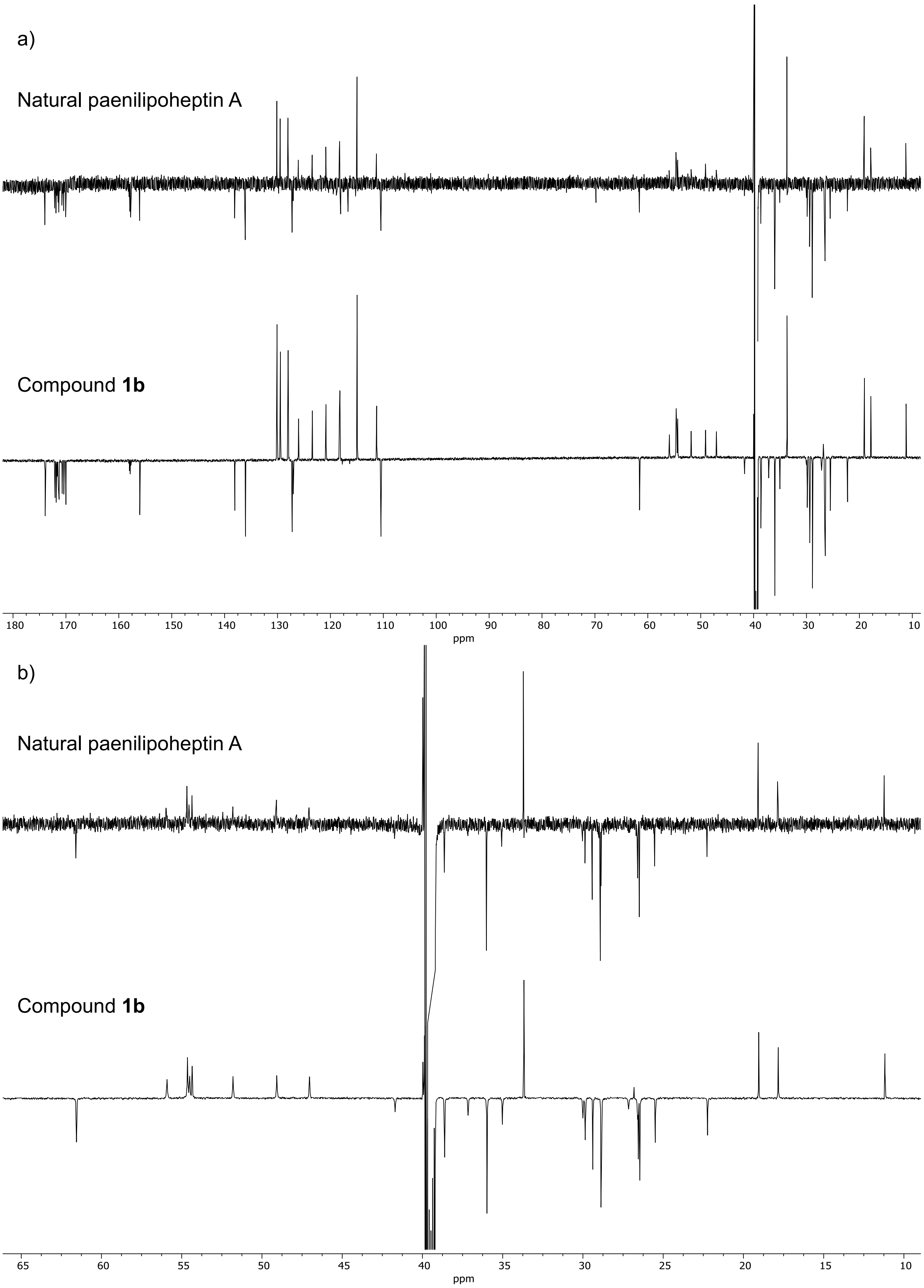

Figure S3. NMR comparison of the ^{13}C-NMR (850 MHz, DMSO-d_6) spectrum of natural paenilipoheptin A isolated after fermentation of the producing organism overlaid with ^{13}C-NMR (850 MHz, DMSO-d_6) spectra of synthetic compound **1b**: a) full spectrum, 180 – 10 ppm region; b) aliphatic carbons region, 65 – 10 ppm

Extended MIC table

Table S2. Minimum inhibitory concentrations (MICs) determined for the natural paenilipoheptin A, compound **1b**, and control antibiotics.

Microorganism/strain tested	MIC (µg/mL)					
	Paenilipoheptin A	**1b**	Vancomycin	Polymyxin B	Iturin A	Nystatin
**Gram-positive bacteria**						
B. subtilis 168	16	8	0.25	ND	ND	ND
E. faecium E980	16	8	0.5	ND	ND	ND
E. faecium VRE E155 (VanA)	ND	8	>128	ND	ND	ND
E. faecium VRE E7314 (VanB)	ND	8	>128	ND	ND	ND
S. aureus ATCC 29213	16	16	0.5	ND	ND	ND
S. aureus USA 300 (MRSA)	16	16	0.5	ND	ND	ND
S. aureus VRS3b	ND	16	>128	ND	ND	ND
S. aureus LIM-2	ND	32	4	ND	ND	ND
Paenibacillus sp. JJ-21	ND	64	0.25	ND	ND	ND
**Gram-negative bacteria**						
E. coli ATCC 25922	ND	>64	ND	2	ND	ND
E. coli BW 25113 *ΔbamB ΔtolC*	ND	>64	ND	1	ND	ND
K. pneumoniae ATCC 13883	ND	>64	ND	1	ND	ND
A. baumannii ATCC 19606	ND	>64	ND	1	ND	ND
P. aeruginosa ATCC 27853	ND	>64	ND	2	ND	ND
**Mycobacteria**						
M. tuberculosis H37Rv	ND	>64	ND	ND	ND	ND
M. smegmatis mc2155	ND	>64	ND	ND	ND	ND
M. abscessus ATCC 19977	ND	>64	ND	ND	ND	ND
**Fungal species**						
S. cerevisiae NSY220.1	ND	>64	ND	ND	32	32
C. albicans ATCC 10231	ND	>64	ND	ND	32	32
P. expansum ATCC 24692	ND	>64	ND	ND	16	4
A. fumigatus Af293	ND	>64	ND	ND	64	32
A. niger N400	ND	>64	ND	ND	16	16
A. oryzae RIB40	ND	>64	ND	ND	64	4
F. oxysporum CBS 101587	ND	>64	ND	ND	64	32

ND: not determined

Hemolysis

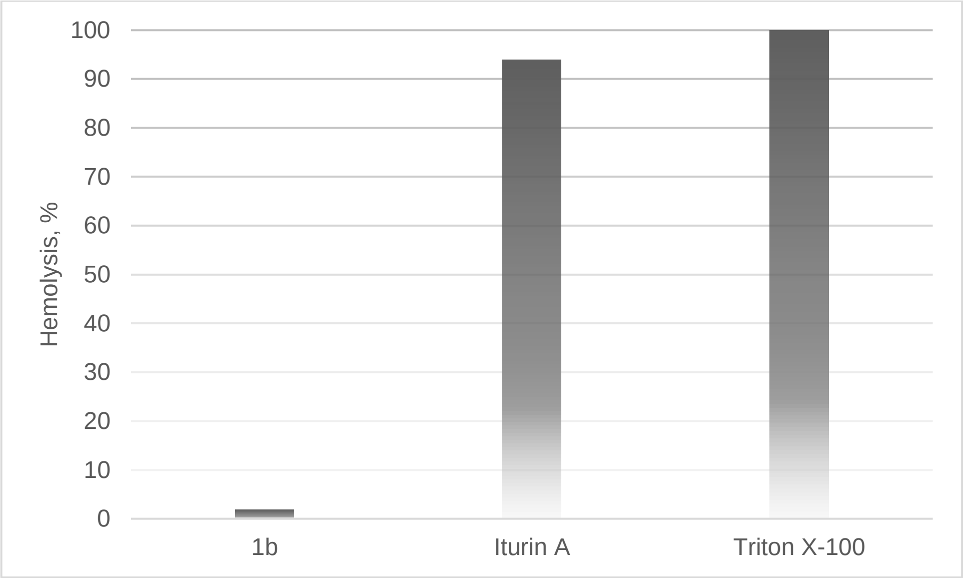

Figure S4. Hemolysis data for the compound **1b** and Iturin A at a concentration of 64 µg/mL after 1 hour. Percentage of the hemolysis: **1b** – 1.9%, Iturin A – 93.9% in comparison to Triton X-100 (100%). The obtained values were used as an average based on n = 3 technical replicates.

NMR Spectra

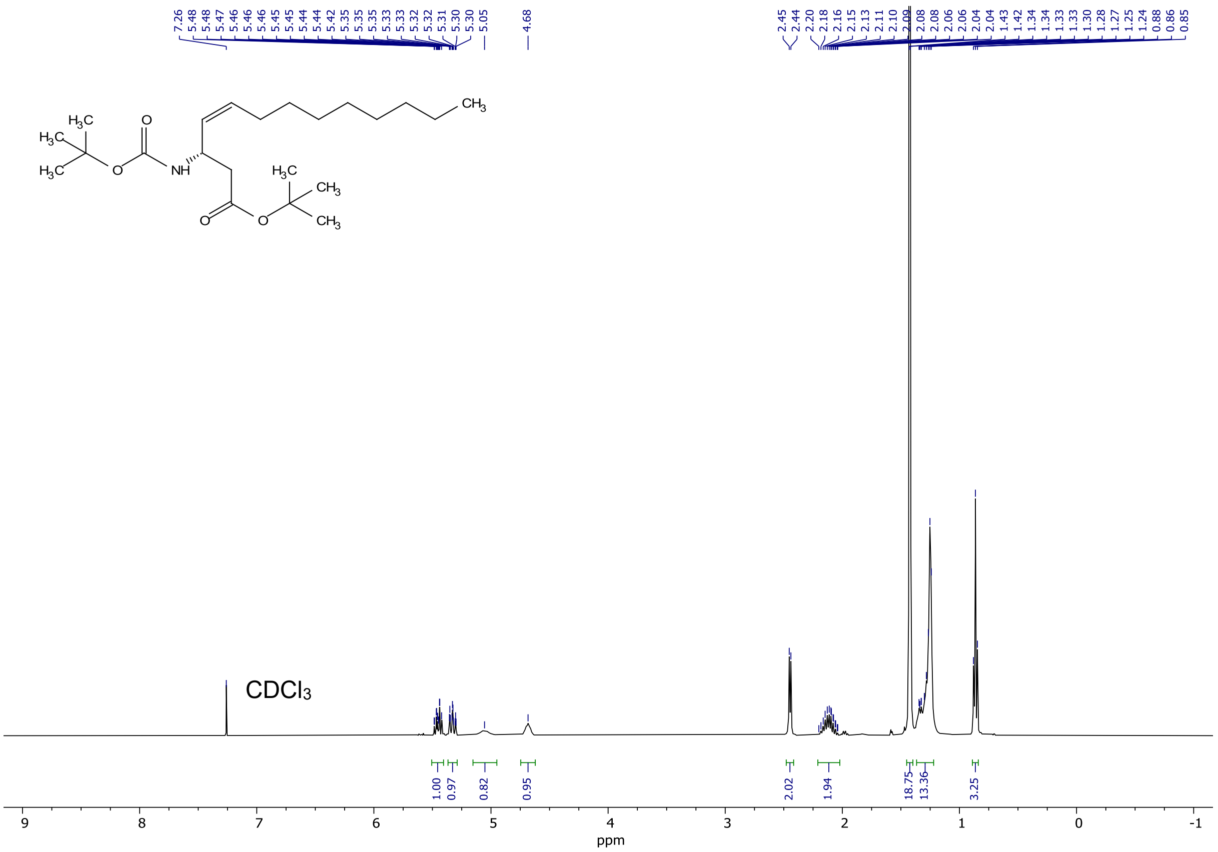

Figure S5. ^{1}H-NMR (400 MHz, $CDCl_3$) of the compound **6a**.

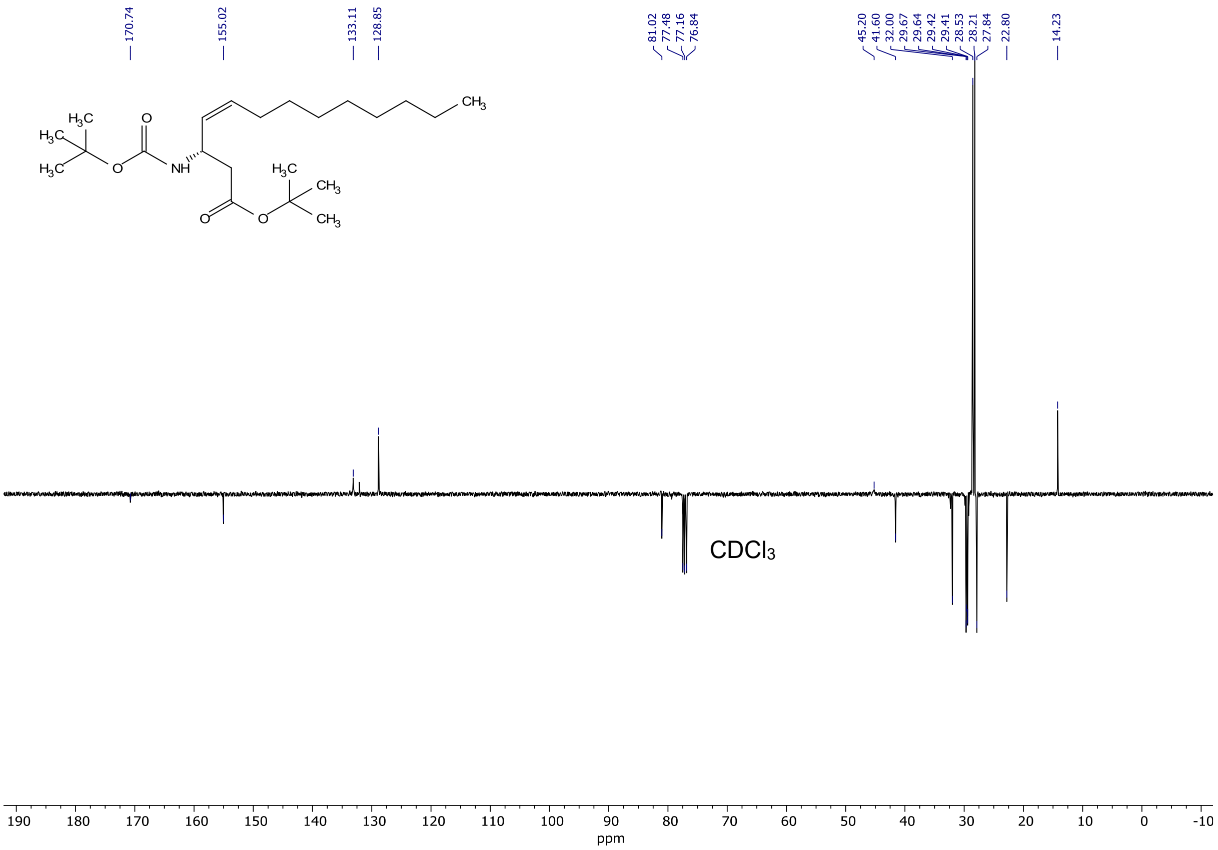

Figure S6. $^{13}C\{^{1}H\}$ NMR (101 MHz, $CDCl_3$) of the compound **6a**.

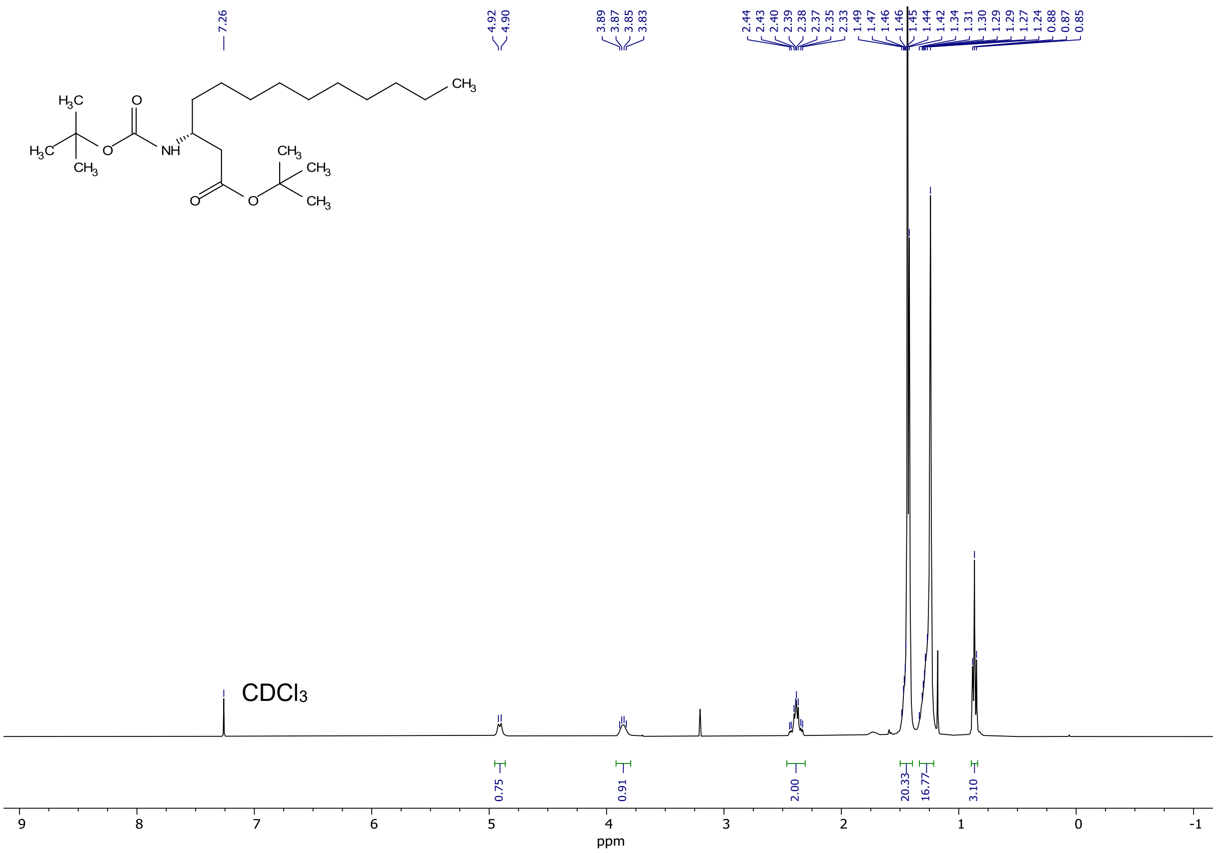

Figure S7. ^{1}H-NMR (400 MHz, $CDCl_3$) of the compound **7a**.

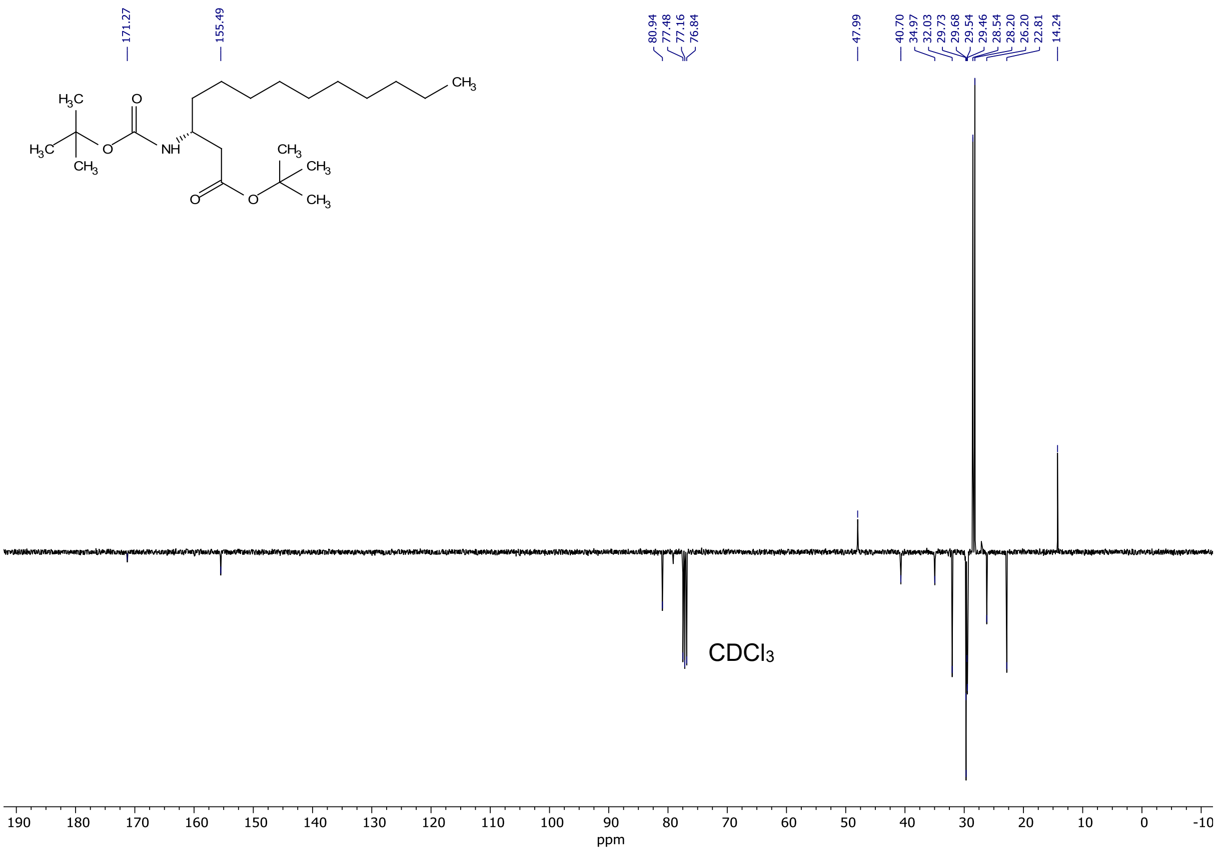

Figure S8. $^{13}C\{^{1}H\}$ NMR (101 MHz, $CDCl_3$) of the compound **7a**.

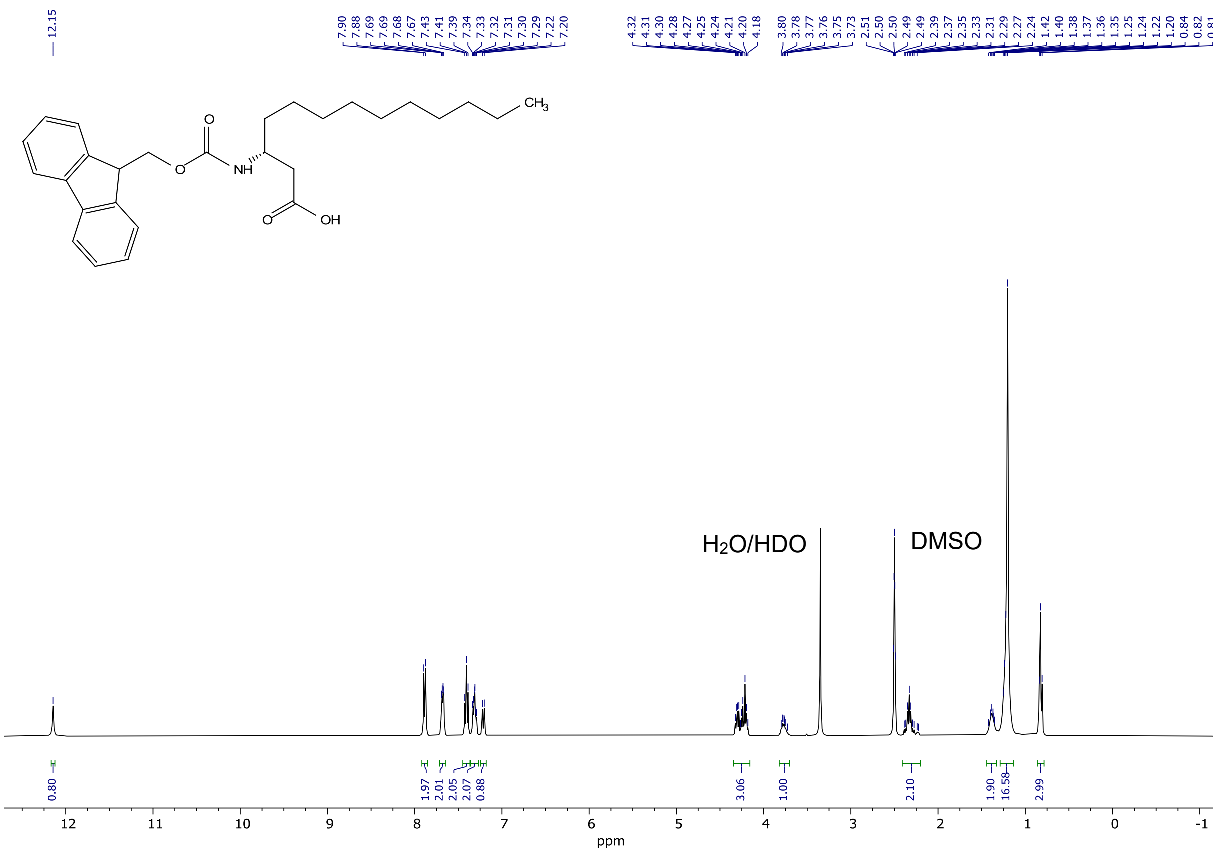

Figure S9. ^{1}H-NMR (400 MHz, DMSO-d_6) of the compound **8a**.

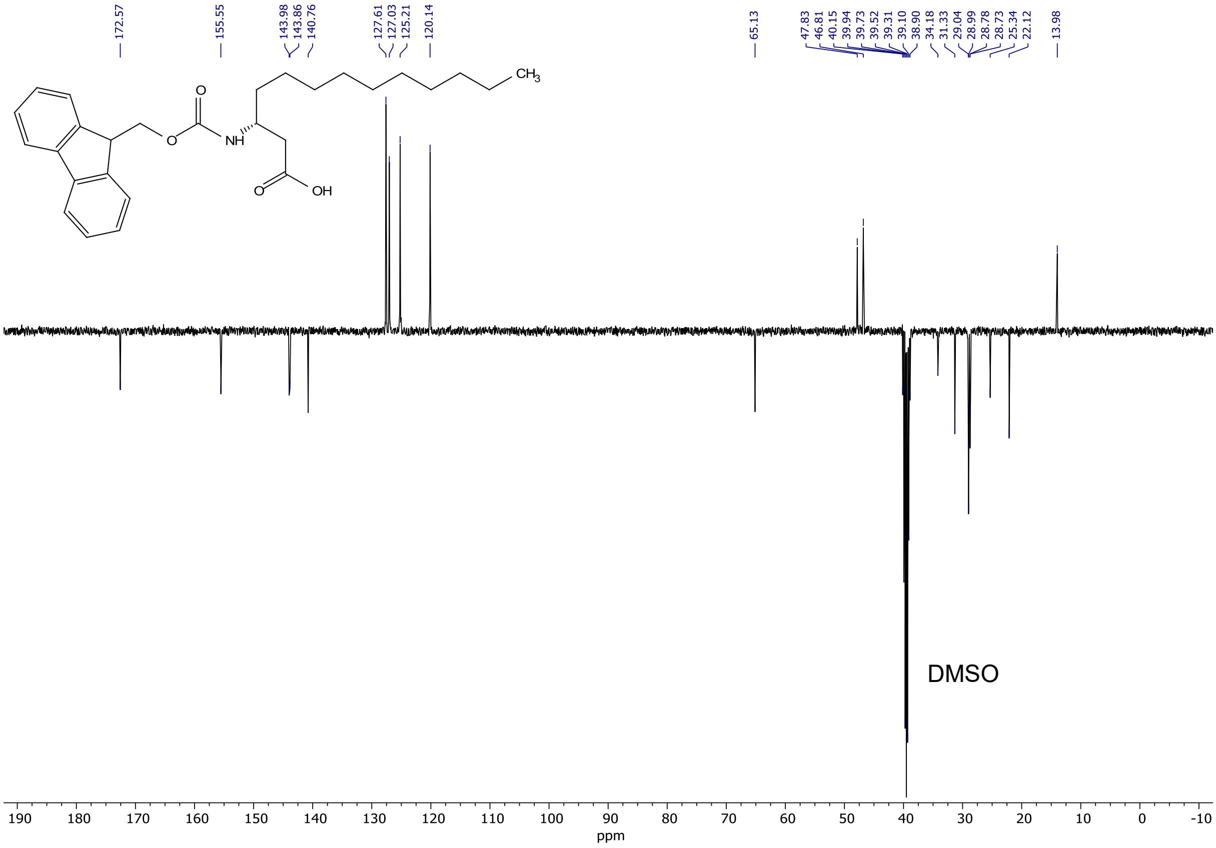

Figure S10. $^{13}C\{^{1}H\}$ NMR (101 MHz, DMSO-d_6) of the compound **8a**.

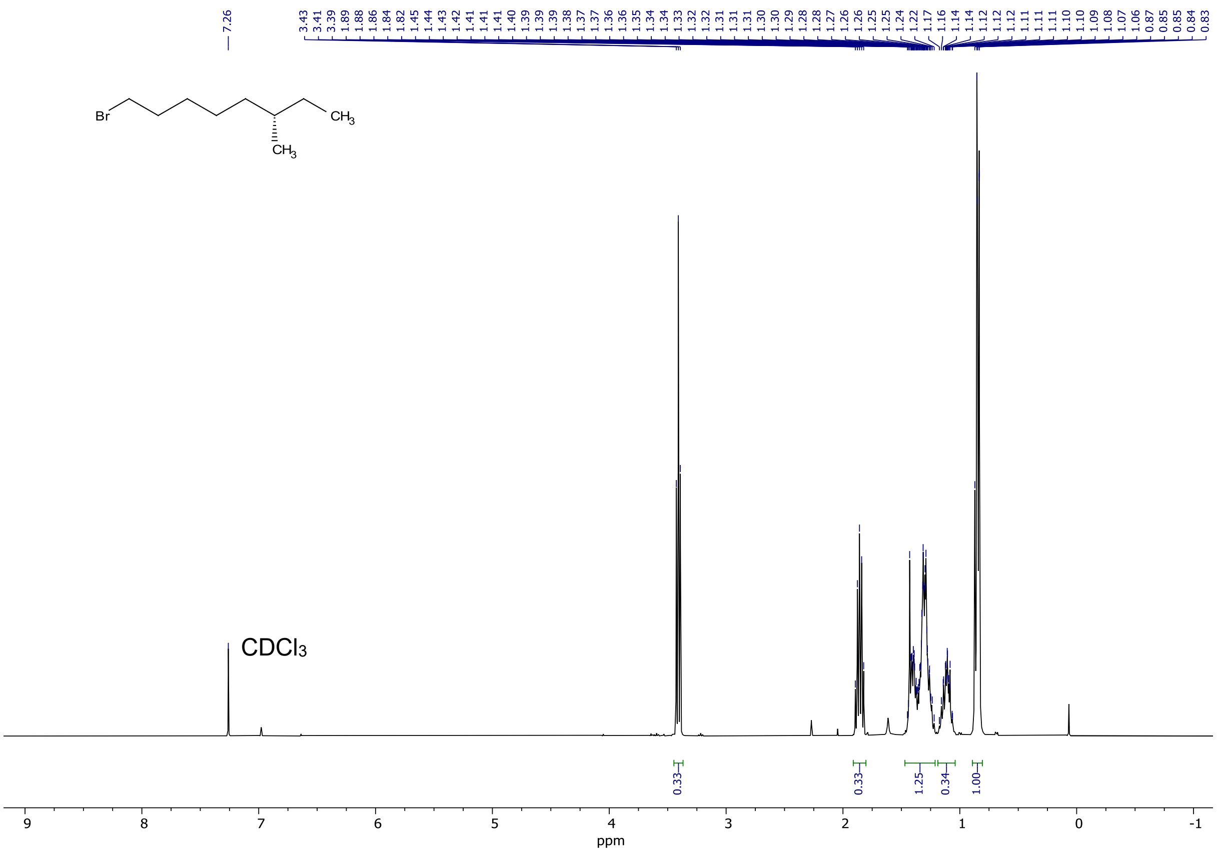

Figure S11. ^{1}H-NMR (400 MHz, $CDCl_3$) of the compound **10**.

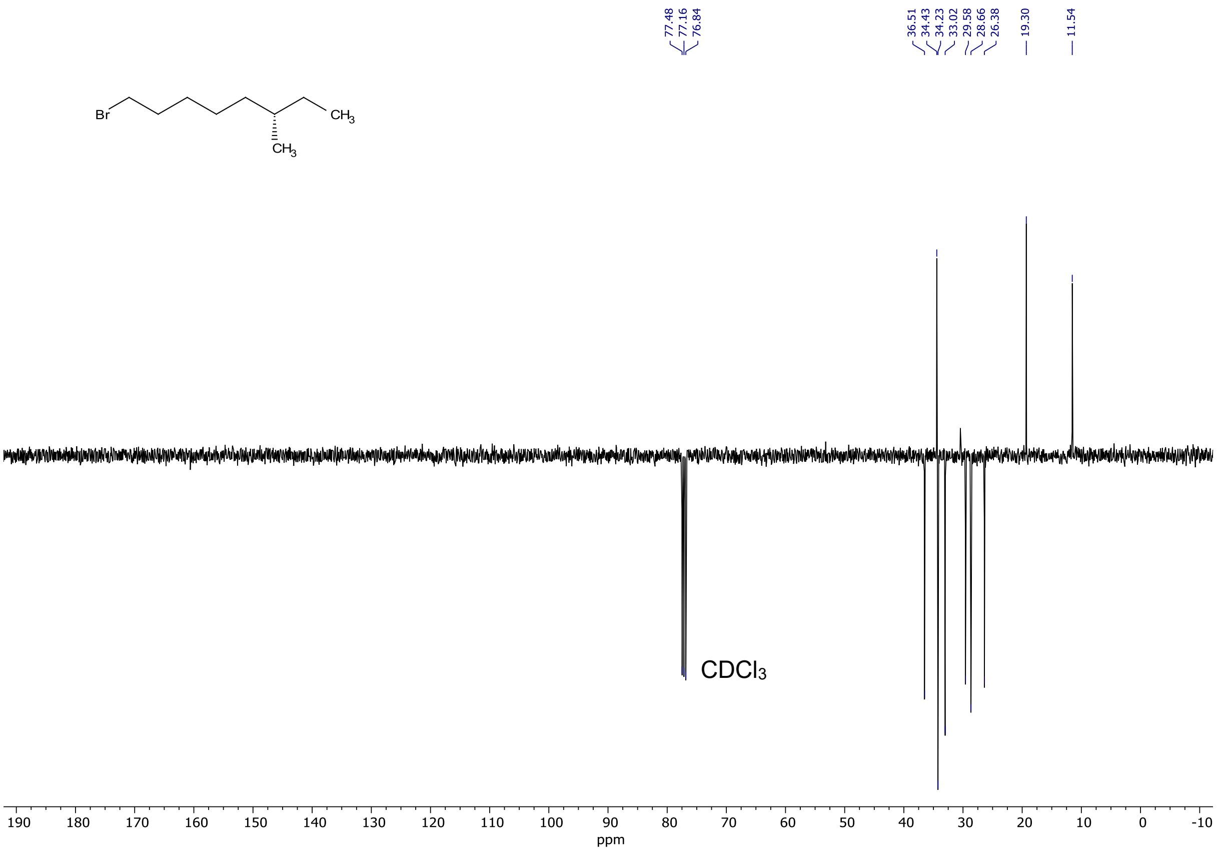

Figure S12. $^{13}C\{^{1}H\}$ NMR (101 MHz, $CDCl_3$) of the compound **10**.

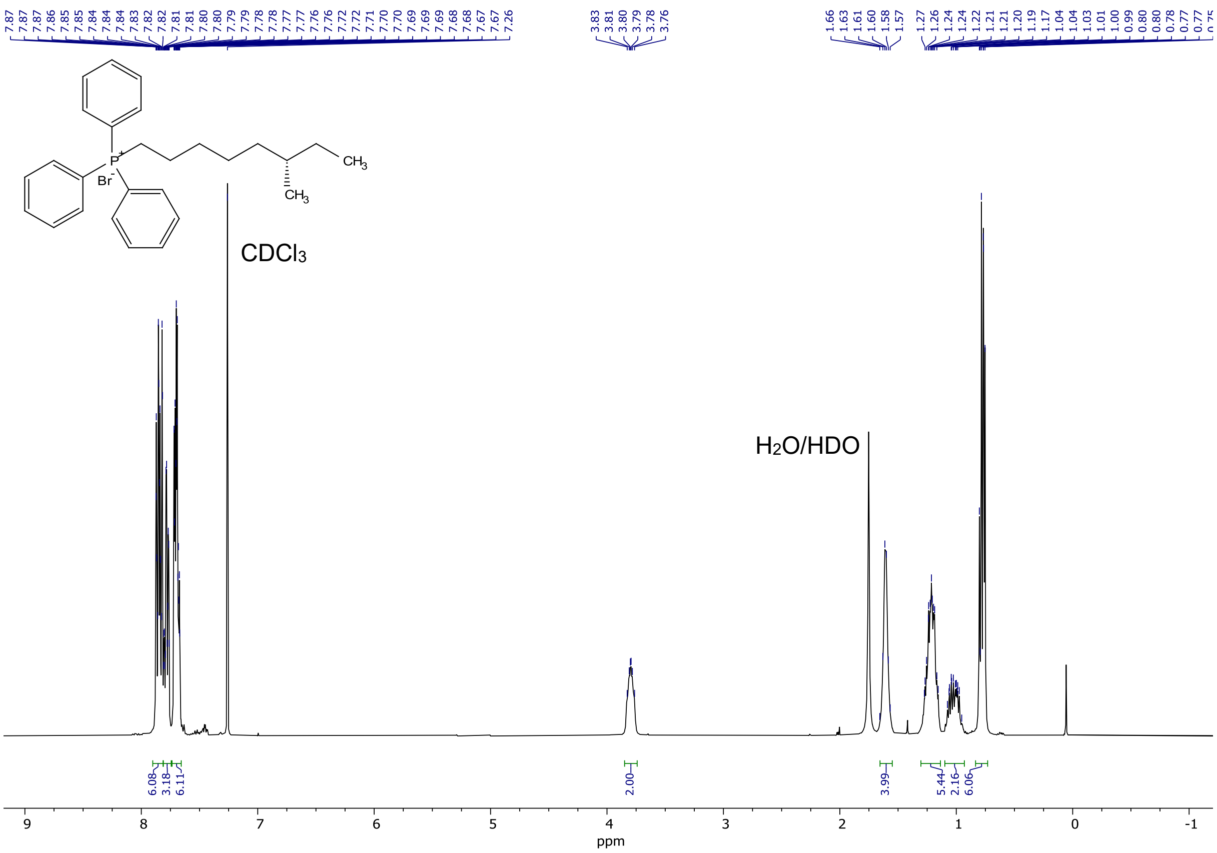

Figure S13. ^{1}H-NMR (400 MHz, $CDCl_3$) of the compound **11**.

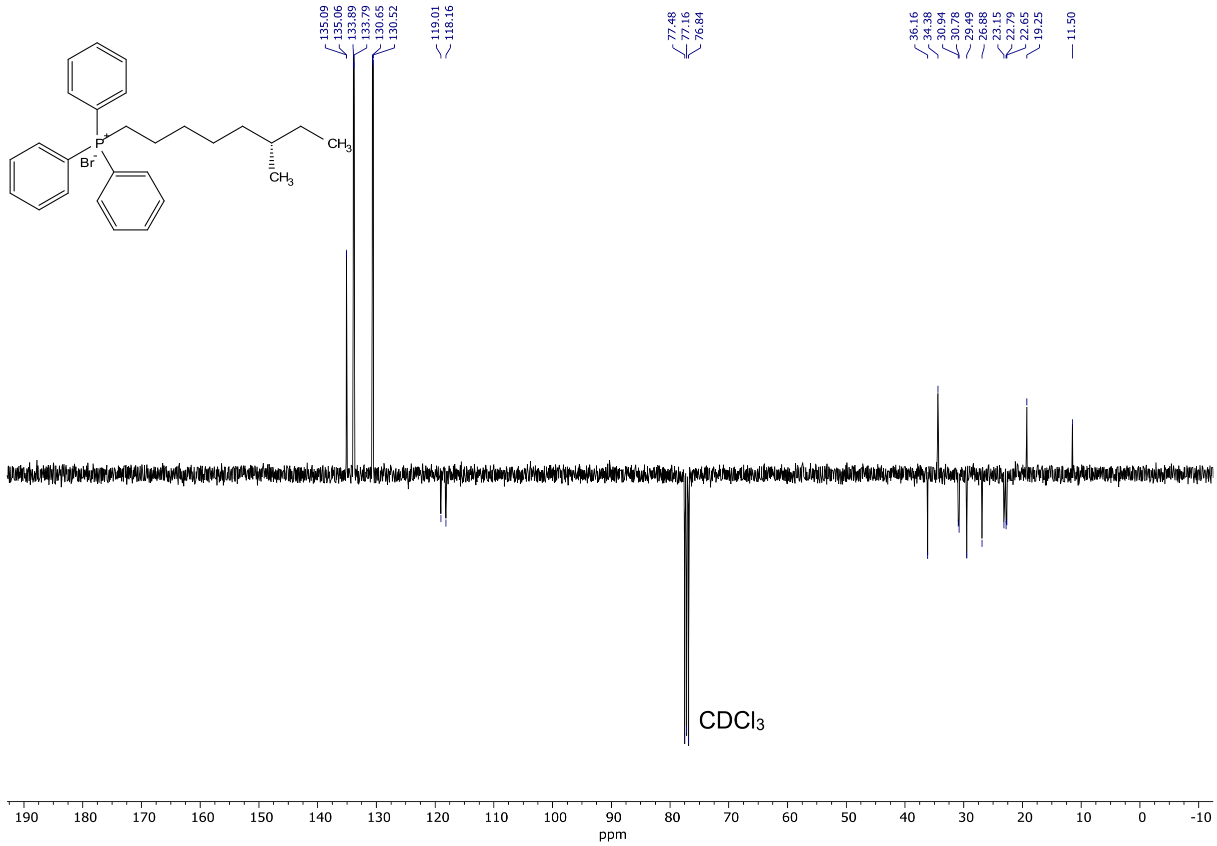

Figure S14. $^{13}C\{^{1}H\}$ NMR (101 MHz, $CDCl_3$) of the compound **11**.

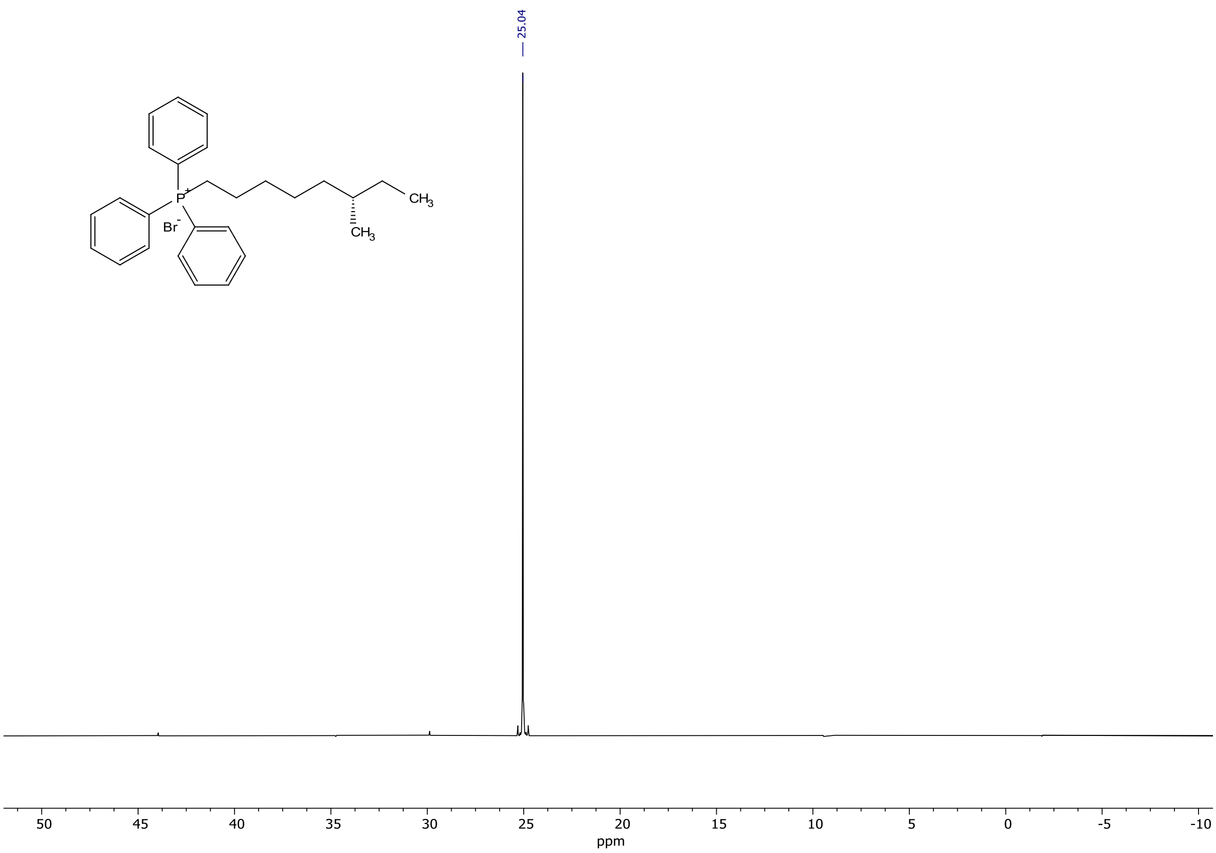

Figure S15. $^{31}P\{^{1}H\}$-NMR (162 MHz, $CDCl_3$) of the compound **11**.

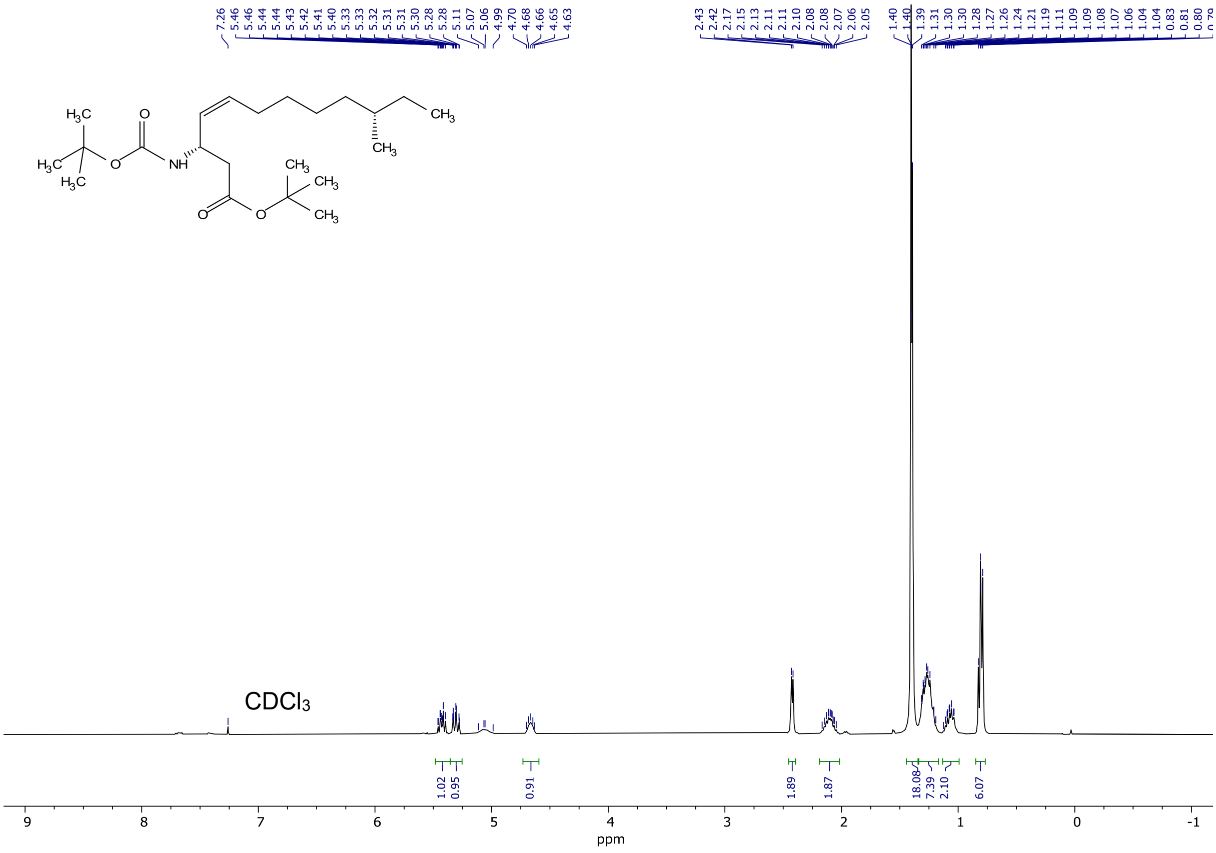

Figure S16. ^{1}H-NMR (400 MHz, $CDCl_3$) of the compound **12**.

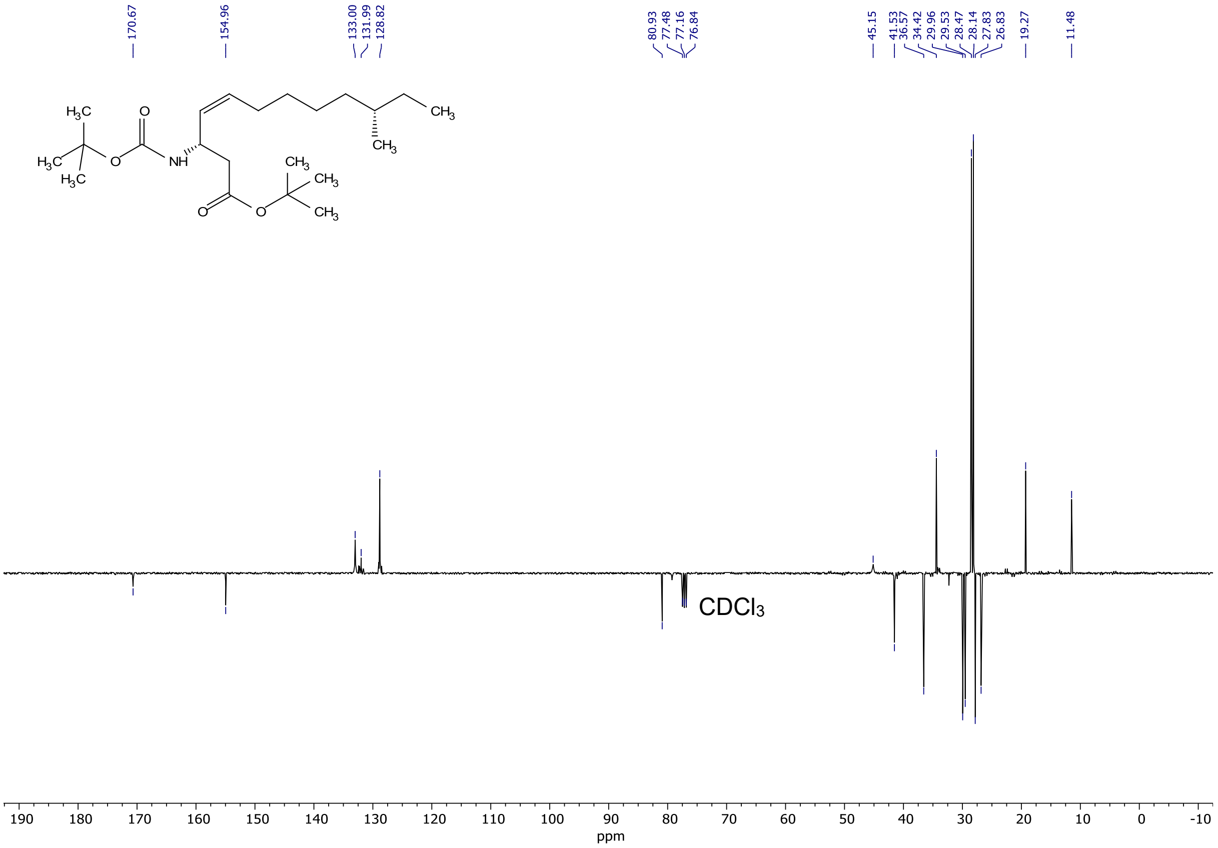

Figure S17. $^{13}C\{^{1}H\}$ NMR (101 MHz, $CDCl_3$) of the compound **12**.

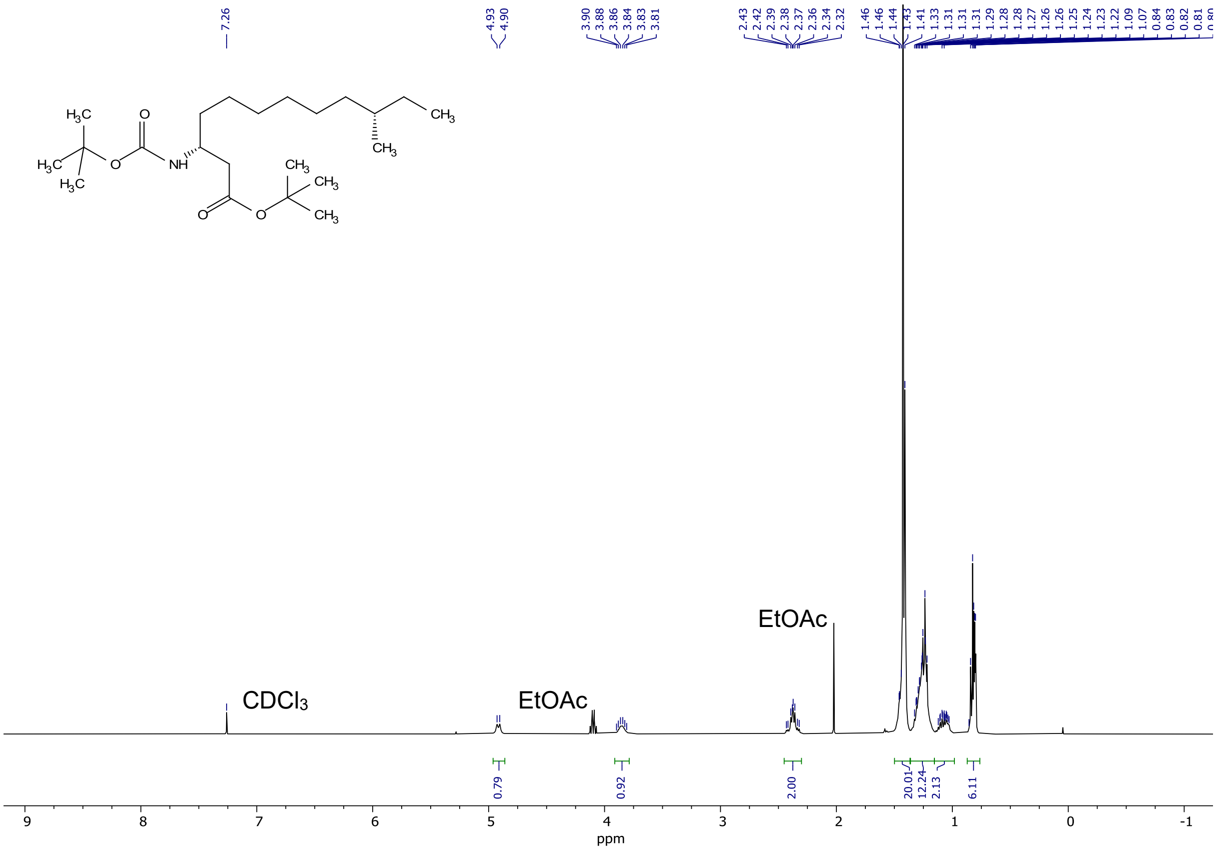

Figure S18. ^{1}H-NMR (400 MHz, $CDCl_3$) of the compound **13**.

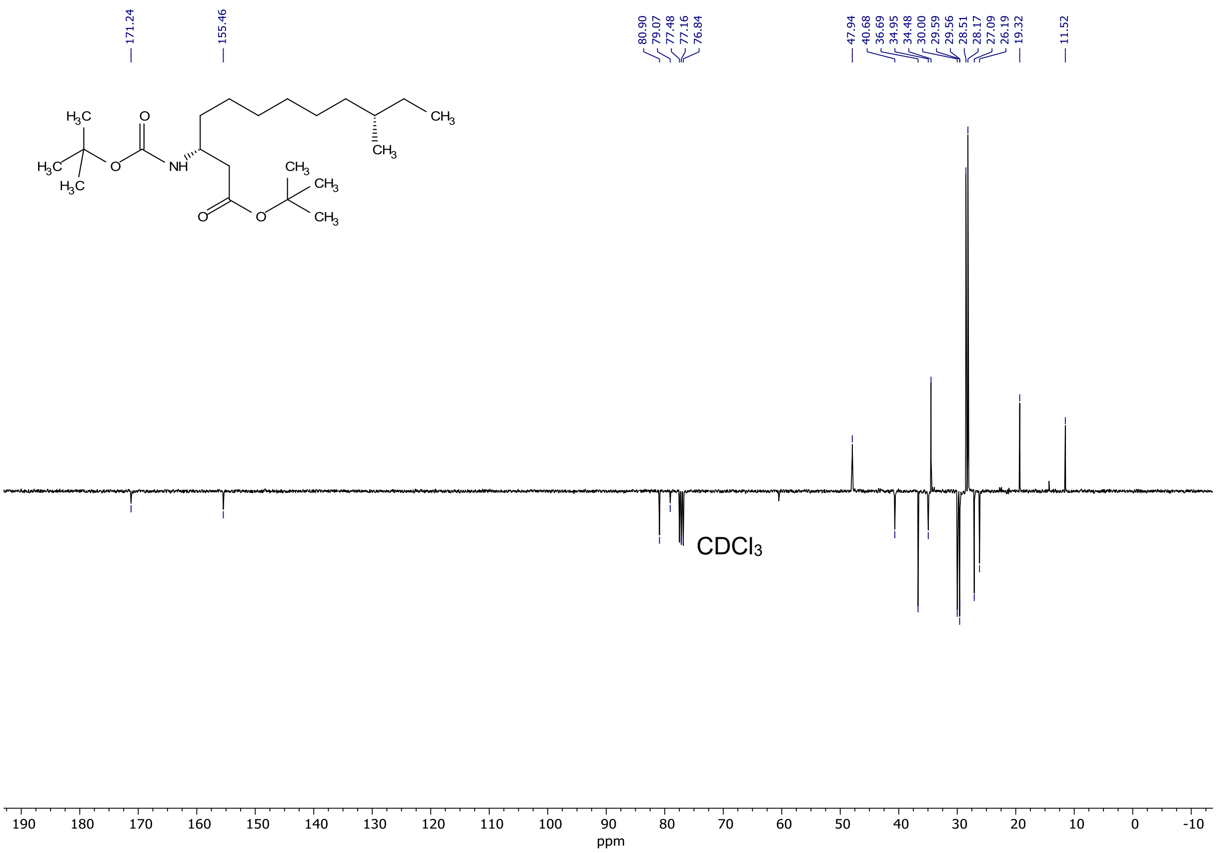

Figure S19. $^{13}C\{^{1}H\}$ NMR (101 MHz, $CDCl_3$) of the compound **13**.

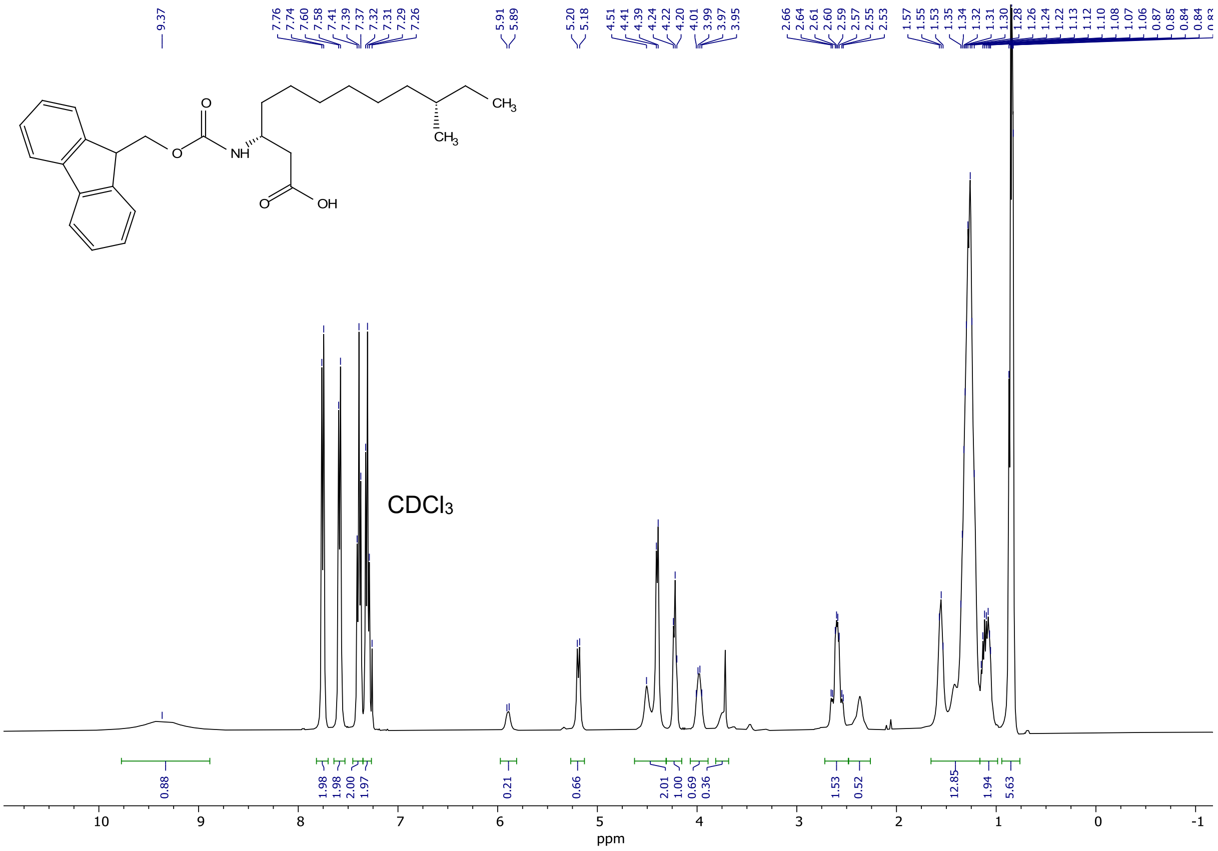

Figure S20. ^{1}H-NMR (400 MHz, $CDCl_3$) of the compound **14**.

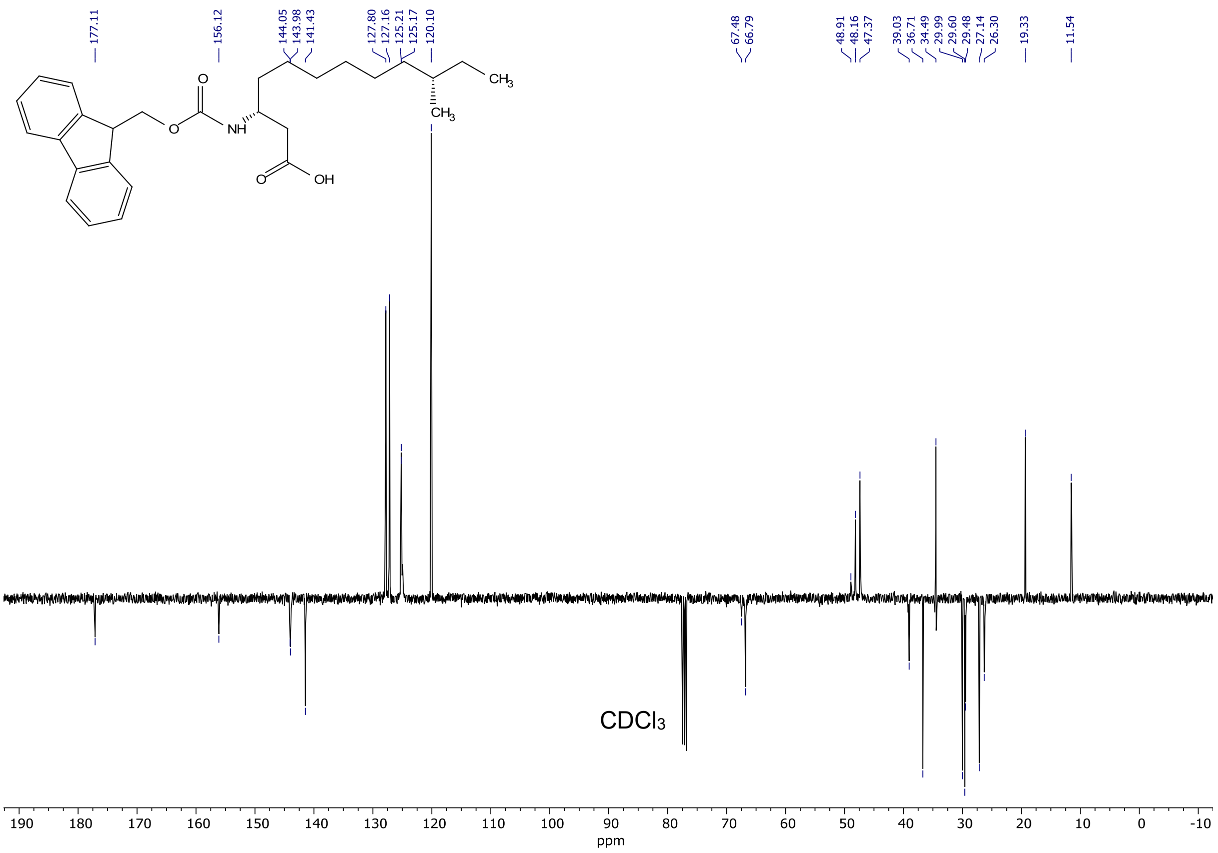

Figure S21. $^{13}C\{^{1}H\}$ NMR (400 MHz, $CDCl_3$) of the compound **14**.

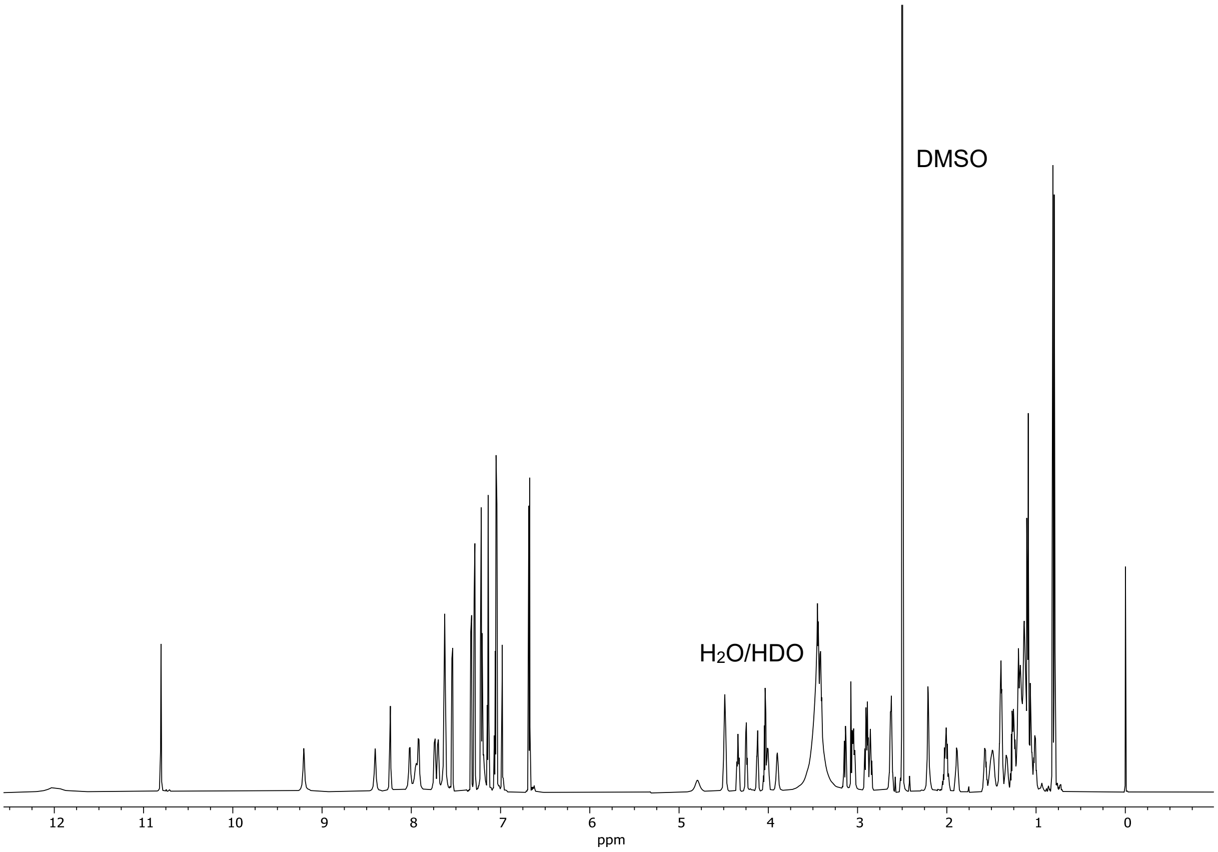

Figure S22. ^{1}H-NMR (850 MHz, DMSO-d_6) of the compound **1b**.

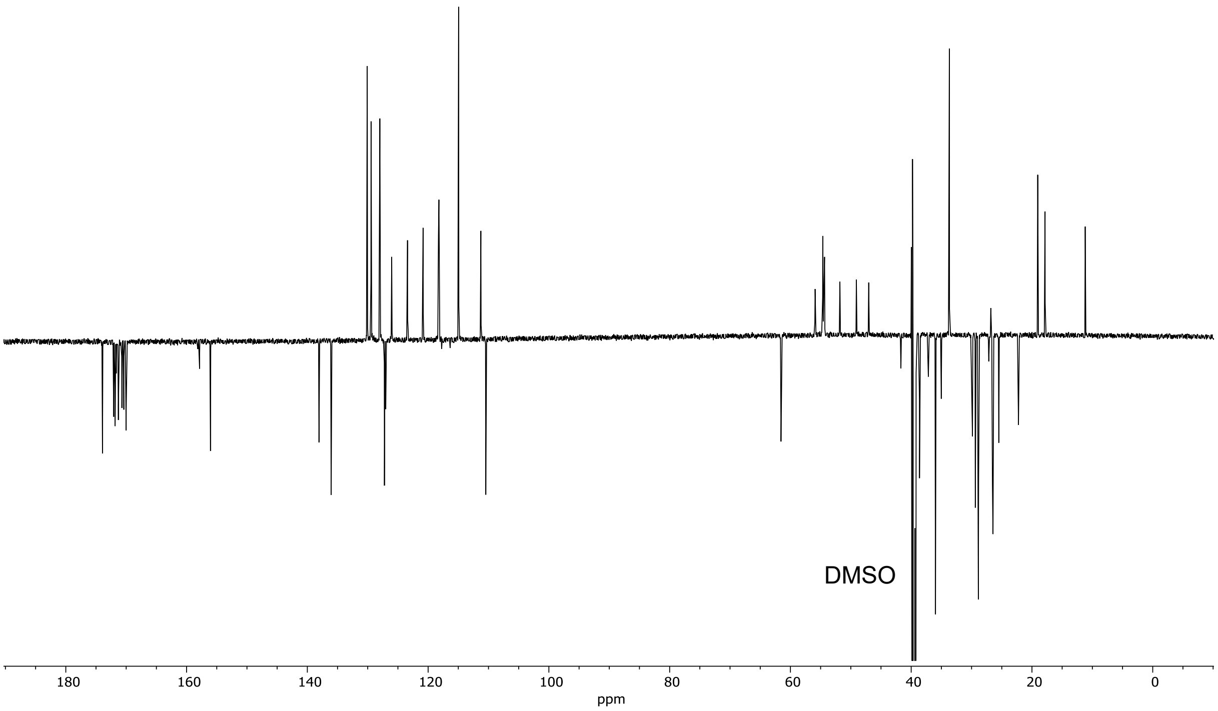

Figure S23. $^{13}C\{^{1}H\}$ NMR (214 MHz, DMSO-d_6) of the compound **1b**.

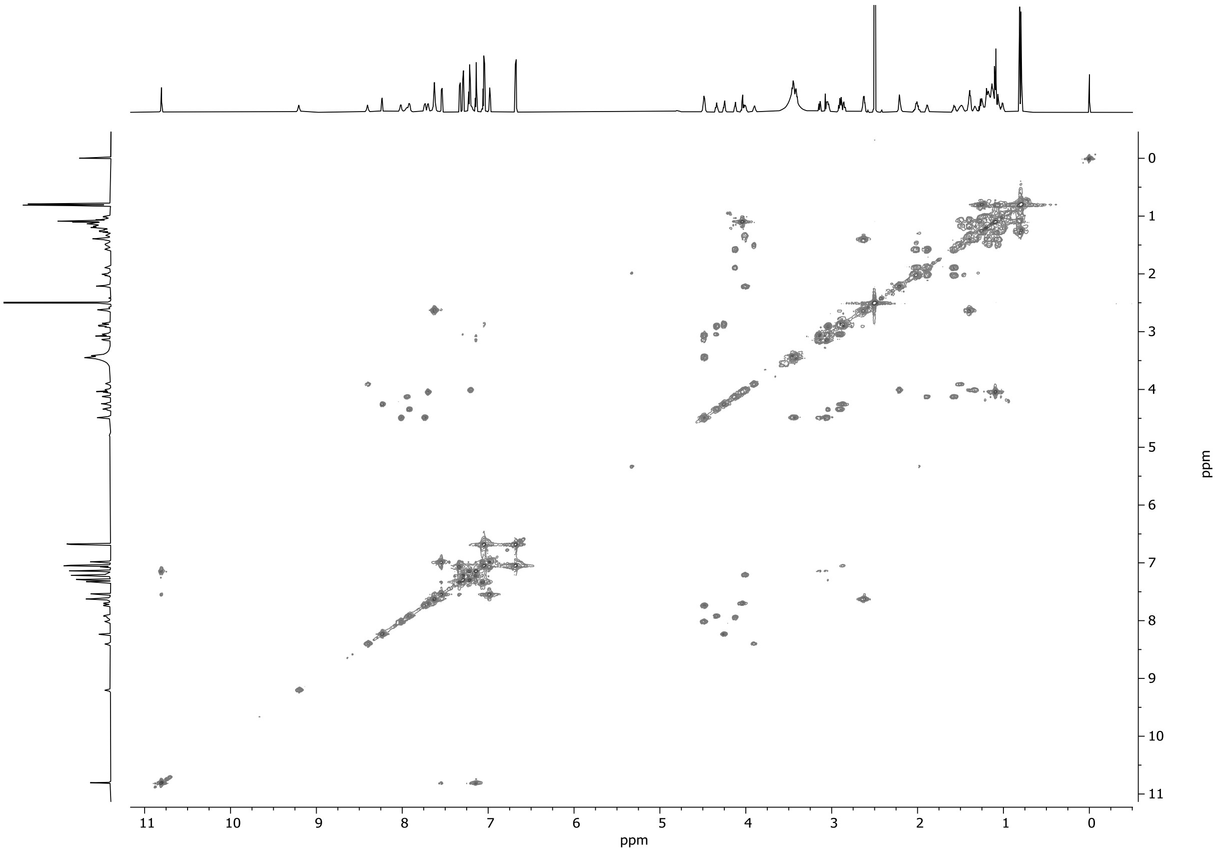

Figure S24. ^{1}H-^{1}H COSY NMR (850 MHz, DMSO-d_6) of the compound **1b**.

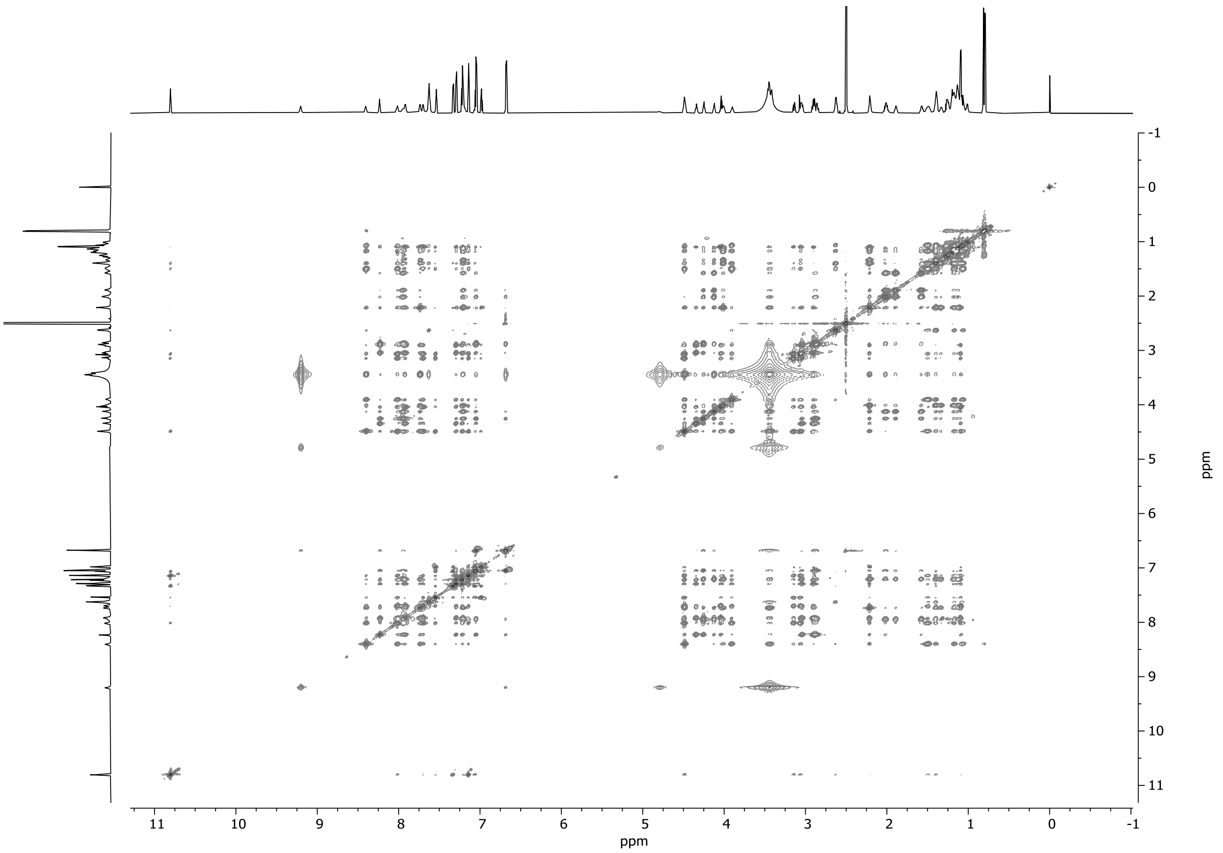

Figure S25. ^{1}H-^{1}H NOESY NMR (850 MHz, DMSO-d_6) of the compound **1b**.

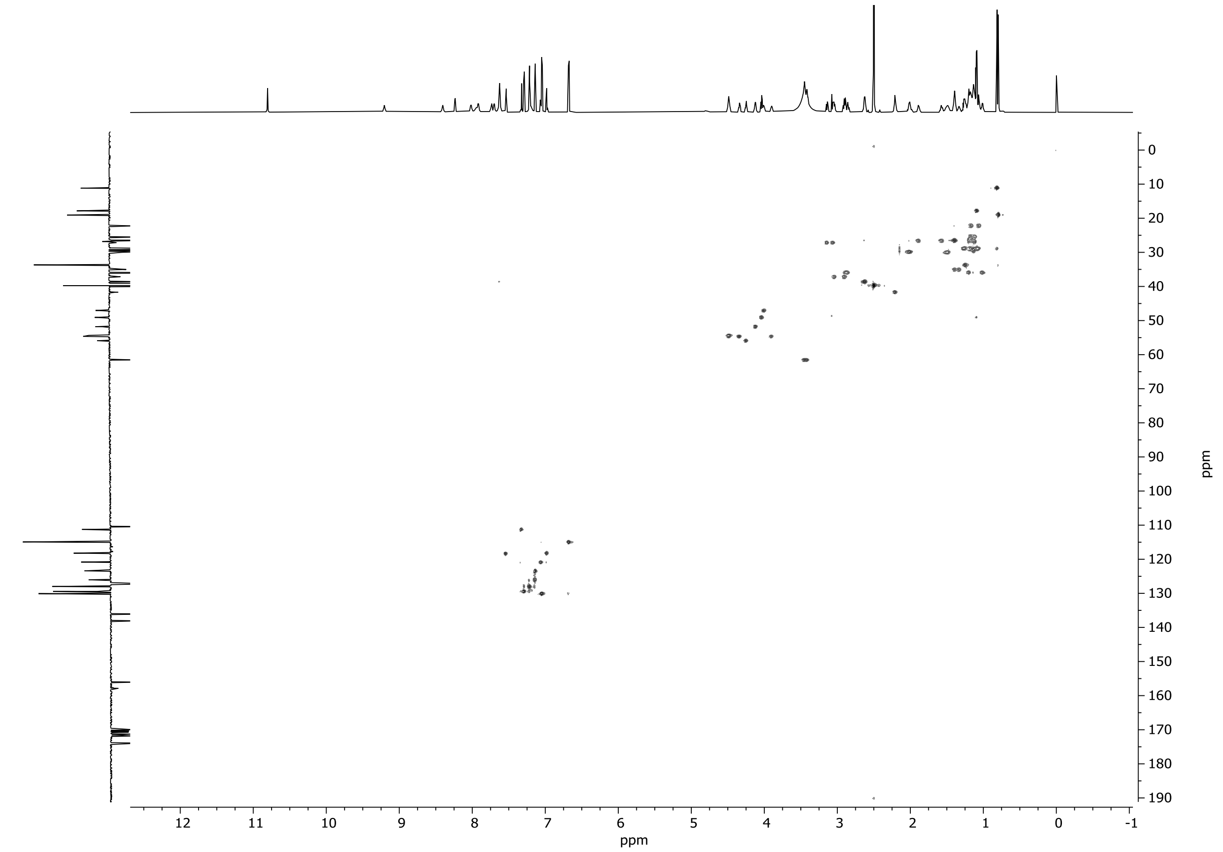

Figure S26. ^{1}H-^{13}C HSQC NMR ((850, 214) MHz, DMSO-d_6) of the compound **1b**.

HPLC analysis of the peptide 1b

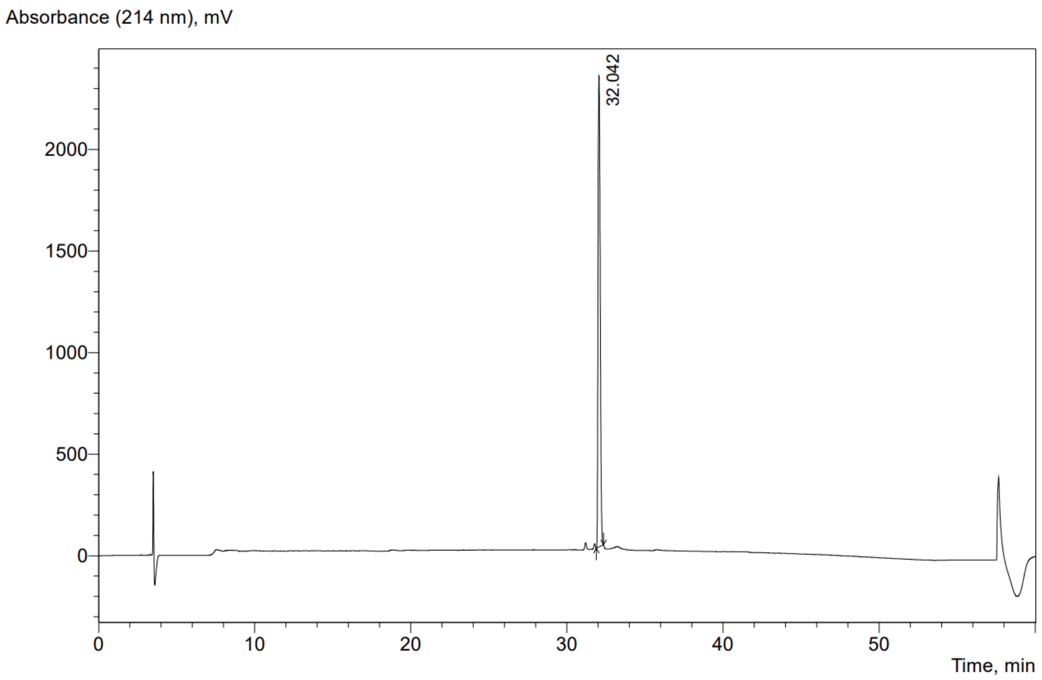

Figure S27. HPLC trace of the purified compound **1b**. The peptide eluted as a single peak at 32.04 min

References

(1) Schmid, R.; Heuckeroth, S.; Korf, A.; Smirnov, A.; Myers, O.; Dyrlund, T. S.; Bushuiev, R.; Murray, K. J.; Hoffmann, N.; Lu, M.; Sarvepalli, A.; Zhang, Z.; Fleischauer, M.; Dührkop, K.; Wesner, M.; Hoogstra, S. J.; Rudt, E.; Mokshyna, O.; Brungs, C.; Ponomarov, K.; Mutabdžija, L.; Damiani, T.; Pudney, C. J.; Earll, M.; Helmer, P. O.; Fallon, T. R.; Schulze, T.; Rivas-Ubach, A.; Bilbao, A.; Richter, H.; Nothias, L. F.; Wang, M.; Orešič, M.; Weng, J. K.; Böcker, S.; Jeibmann, A.; Hayen, H.; Karst, U.; Dorrestein, P. C.; Petras, D.; Du, X.; Pluskal, T. Integrative Analysis of Multimodal Mass Spectrometry Data in MZmine 3. *Nature Biotechnology* **2023**, pp 447–449. DOI: 10.1038/s41587-023-01690-2.

(2) Henry, L.; Leung, I. K. H.; Claridge, T. D. W.; Schofield, C. J. γ-Butyrobetaine Hydroxylase Catalyses a Stevens Type Rearrangement. *Bioorg. Med. Chem. Lett.* **2012**, *22* (15), 4975–4978. DOI: 10.1016/j.bmcl.2012.06.024.

(3) Patil, S. M.; Rao Dumpala, R. M.; Goswami, D.; Dawar, R.; Gupta, R. Mechanistic Approach to Reveal Interaction of Uranyl Ions in Alkyltriphenylphosphonium Bromide-Based Deep Eutectic Solvent. *Inorg. Chem.* **2022**, *61* (32), 12599–12609. DOI: 10.1021/acs.inorgchem.2c01547.

(4) Řezanka, T.; Sigler, K. Volatile Lactones – (5*S*, *S*)-5-Methyl-3-(Methylalkyl)Furan-2(5 *H*)-ones – Identified in the Submerged Cultivation of *Streptomyces Avermitilis*. *Eur. J. Org. Chem.* **2006**, *2006* (18), 4277–4284. DOI: 10.1002/ejoc.200600343.

(5) Marfey, P. Determination OfD-Amino Acids. II. Use of a Bifunctional Reagent, 1,5-Difluoro-2,4-Dinitrobenzene. *Carlsberg Res. Commun.* **1984**, *49* (6), 591–596. DOI: 10.1007/BF02908688.

(6) Vijayasarathy, S.; Prasad, P.; Fremlin, L. J.; Ratnayake, R.; Salim, A. A.; Khalil, Z.; Capon, R. J. C3 and 2D C3 Marfey's Methods for Amino Acid Analysis in Natural Products. *J. Nat. Prod.* **2016**, *79* (2), 421–427. DOI: 10.1021/acs.jnatprod.5b01125.
